# Multi-modality in gene regulatory networks with slow promoter kinetics

**DOI:** 10.1371/journal.pcbi.1006784

**Published:** 2019-02-19

**Authors:** M. Ali Al-Radhawi, Domitilla Del Vecchio, Eduardo D. Sontag

**Affiliations:** 1 Departments of Electrical and Computer Engineering and of Bioengineering, Northeastern University, Boston, Massachusetts, United States of America; 2 Department of Mechanical Engineering, Massachusetts Institute of Technology, Cambridge, Massachusetts, United States of America; 3 Laboratory of Systems Pharmacology, Harvard Medical School, Boston, Massachusetts, United States of America; University of Illinois at Urbana-Champaign, UNITED STATES

## Abstract

Phenotypical variability in the absence of genetic variation often reflects complex energetic landscapes associated with underlying gene regulatory networks (GRNs). In this view, different phenotypes are associated with alternative states of complex nonlinear systems: stable attractors in deterministic models or modes of stationary distributions in stochastic descriptions. We provide theoretical and practical characterizations of these landscapes, specifically focusing on stochastic Slow Promoter Kinetics (SPK), a time scale relevant when transcription factor binding and unbinding are affected by epigenetic processes like DNA methylation and chromatin remodeling. In this case, largely unexplored except for numerical simulations, adiabatic approximations of promoter kinetics are not appropriate. In contrast to the existing literature, we provide rigorous analytic characterizations of multiple modes. A general formal approach gives insight into the influence of parameters and the prediction of how changes in GRN wiring, for example through mutations or artificial interventions, impact the possible number, location, and likelihood of alternative states. We adapt tools from the mathematical field of singular perturbation theory to represent stationary distributions of Chemical Master Equations for GRNs as mixtures of Poisson distributions and obtain explicit formulas for the locations and probabilities of metastable states as a function of the parameters describing the system. As illustrations, the theory is used to tease out the role of cooperative binding in stochastic models in comparison to deterministic models, and applications are given to various model systems, such as toggle switches in isolation or in communicating populations, a synthetic oscillator, and a trans-differentiation network.

## Author summary

Regulatory mechanisms of slow gene activation and deactivation play a role in triggering and sustaining phenotypically heterogeneous, yet genetically identical (clonal), cellular populations in a wide variety of biological processes. These range from embryonic development and hematopoietic cell differentiation to the emergence of tumor heterogeneity and consequent resistance to therapy. In contrast to previously reported numerical simulations, we introduce in this paper a theoretical and computational approach to the characterization of the multi-attractor dynamic landscape of gene networks with slow promoter kinetics. We obtain precise formulas that are then illustrated through applications to several systems biology models including a trans-differentiation network and a communicating population of synthetic toggle switches.

## Introduction

A gene regulatory network (GRN) consists of a collection of genes that transcriptionally regulate each other through their expressed proteins. Through these interactions, including positive and negative feedback loops, GRNs play a central role in the overall control of cellular life [1–3]. The behavior of such networks is stochastic due to the random nature of transcription, translation, and post-translational protein modification processes, as well as the varying availability of cellular components that are required for gene expression. Stochasticity in GRNs is a source of phenotypic variation among genetically identical (clonal) populations of cells or even organisms [4], and is considered to be one of the mechanisms facilitating cell differentiation and organism development [5]. This phenotypic variation may also confer a population an advantage when facing fluctuating environments [6, 7]. Stochasticity due to randomness in cellular components and transcriptional and translational processes have been thoroughly researched [8, 9].

The fast equilibration of random processes sometimes allows stochastic behavior to be “averaged out” through the statistics of large numbers at an observational time-scale, especially when genes and proteins are found in large copy numbers. In those cases, an entire GRN, or portions of it, might be adequately described by a deterministic model. Stochastic effects that occur at a slower time scale, however, may render a deterministic analysis inappropriate and might alter the steady-state behavior of the system. This paper addresses a central question about GRNs: how many different “stable steady states” can such a system potentially settle upon, and how does stochasticity, or lack thereof, affect the answer? To answer this question, it is necessary to understand the possibly different predictions that follow from stochastic versus deterministic models of gene expression. Indeed, qualitative conclusions regarding the steady-state behavior of gene expression levels in a GRN are critically dependent on whether a deterministic or stochastic model is used (see [10] for a recent review). It follows that the mathematical characterization of phenomena such as non-genetic phenotype heterogeneity, switching behavior in response to environmental conditions, and lineage conversion in cells, will depend on the choice of the model.

In order to make the discussion precise, we must clarify the meaning of the term “stable steady state” in both the deterministic and stochastic frameworks. Deterministic models are employed when molecular concentrations are large, or if stochastic effects can be averaged out. They consist of systems of ordinary differential equations describing averaged-out approximations of the interactions between the various molecular species in the GRN under study. For these systems, steady states are the zeroes of the vector field defining the dynamics, and “stable” states are those that are locally asymptotically stable. The number of such stable states quantifies the degree of “multi-stability” of the system. Stochastic models of GRNs, in contrast, are based upon continuous-time Markov chains which describe the random evolution of discrete molecular count numbers. Their long-term behavior is characterized by a stationary Probability Mass Function (PMF) that describes the gene activity configurations and the protein numbers recurrently visited. Under weak ergodicity assumptions, this stationary PMF is unique [11], so multi-stability in the sense of multiple steady states of the Markov chain (MC) is not an interesting notion. A biologically meaningful notion of “multi-stability” in this context, and the one that we employ in our study, is “multi-modality,” meaning the existence of multiple modes (local maxima) of stationary PMFs.

Intuitively, given a multi-stable deterministic system, adding noise may help to “shake” states, dislodging them from one basin of attraction of one stable state, and sending them into the basin of attraction of another stable state. Therefore, in the long run, we are bound to see the various deterministic stable steady states with higher probability, that is to say, we expect that they will appear as modes in the stationary PMF of the MC of the associated stochastic model. This is indeed a typical way in which modes can be interpreted as corresponding to stable states, with stochasticity responsible for the transitions between multiple stable states [12]. However, new modes could arise in the stationary PMF of a stochastic system besides those associated with stable states of the deterministic model, and this can occur even if the deterministic model had just a single stable state. This phenomenon of “stochastic multi-stability” has attracted considerable attention lately, both in theoretical and experimental work [8, 9, 13–15]. Stochastic multi-stability has been linked to behaviors such as transcriptional bursting/pulsing [16, 17] and GRN’s binary response [18]. Furthermore, multi-state gene transcription [4] has been used to propose explanations for phenotypic heterogeneity in isogenic populations.

A common assumption in gene regulation models is that transcription factor (TF) to gene binding/unbinding is significantly faster than the rate of protein production and decay [1]. However, it has been proposed [9, 19] that the emergence of new modes in stochastic systems in addition to those that arise from the deterministic model might be due to low gene copy numbers and *Slow Promoter Kinetics* (SPK), which means that the process of binding and unbinding of TFs to promoters is slow. Thus, the emergence of multi-modality may be due to the slow TF-gene binding and unbinding. Already in prokaryotic cells, where DNA is more accessible to TF binding than in eukaryotic cells, some transcription factors can take several minutes to find their targets, comparable or even higher than the time required for gene expression [20], [21], [22]. This is more relevant in eukaryotic cells, in which transcriptional regulation is often mediated by an additional regulation layer dictated by DNA methylation and histone modifications, commonly referred to as chromatin dynamics. For example, the presence of nucleosomes makes binding sites less accessible to TFs and therefore TF-gene binding/unbinding is modulated by the process of chromatin opening [23], [9, 24–26]. DNA methylation, in particular, has also been reported to slow down TF-gene binding/unbinding [27]. Several experiments have consolidated the role of the aforementioned complex transcription processes in SPK [17, 27–29].

**Fig 1:**
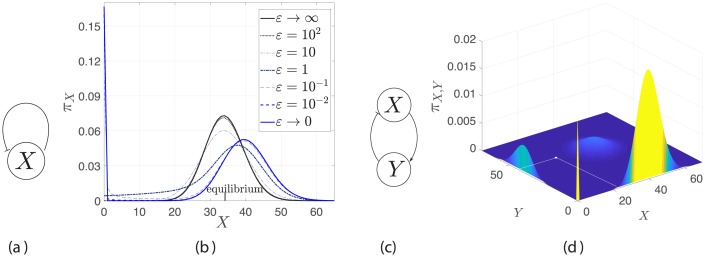
Emergence of multi-modality due to SPK. (a) A diagram of a self-repressing gene, where *ε* is a parameter that multiplies the kinetic rates of all gene reactions (b) The stationary PMF for different *ε* which is showing transition from fast promoter kinetics, i.e., *ε* → ∞, to SPK, i.e., *ε* → 0, in a non-cooperative self-repressing gene. The stationary PMF is bimodal for small *ε* and unimodal for large *ε*. The deterministic equilibrium coincides with the fast kinetics mode. Refer to S1 Text §6.1. (c) A diagram of a repression-activation two-node network. (d) SPK gives rise to four modes while the deterministic model admits a unique stable equilibrium which is marked as a white point, refer to S1 Text §6.2. The surface is plotted using (10).

In summary, new modes may appear in the stationary PMF that do not correspond to stable states in the deterministic model. Conversely, multiple steady states in the deterministic model may collapse, being “averaged out” by noise, with a single mode representing their mean. It is a well-established fact that, in general, multi-stability of the deterministic description of a biochemical network and multi-modality of the associated stochastic model do not follow from each other [30]. This is especially true in low copy number regimes with SPK. Fig 1 gives two examples for the emergence of new modes due to SPK, and it shows that equilibria derived from the corresponding deterministic model do not provide relevant information on the number and locations of the modes.

Here, we pursue a mathematical analysis of the role of SPK in producing multi-modality in GRNs and show analytically how the shape of the stationary PMF is dictated by key biochemical parameters. Previous studies of the Chemical Master Equation (CME) for single genes have already observed bimodality emerging with slow TF-gene binding/unbinding [19, 31–34]. This phenomenon was also studied by taking the limit of SPK using the linear noise approximation [35], linear mapping approximation [36], or hybrid stochastic models of gene expression [37, 38]. The unregulated gene (S1 Text §3.1) has been validated for transcriptional bursting [17]. However, and despite its application relevance, mathematical analysis of the CME for multi-gene networks with SPK has been missing, and only numerical solutions have been reported [39, 40].

In this work, an underlying theoretical contribution is the partitioning of the state space into weakly-coupled ergodic classes [11] which, in the limit of slow binding/unbinding, results in the reduction of the infinite-dimensional MC into a finite-dimensional MC whose states correspond to “promoter states”. In this limit, the stationary PMF of the network can be expressed as a mixture of Poisson distributions, each corresponding to conditioning the MC on a certain promoter configuration. The framework proposed here enables us to analytically determine how the number of modes, their locations, and weights depend on the biophysical parameters. Hence, the proposed framework can be applied to GRNs to predict the different phenotypes that the network can exhibit with low gene copy numbers and SPK.

The results are derived by introducing a new formalism to model GRNs with arbitrary numbers of genes, based on continuous-time MCs. Then, we analyze the stationary solution of the associated CME through a systematic application of the method of singular perturbations [41]. Specifically, we study the SPK limit by letting the ratio of kinetic rate constants of the TF-gene binding/unbinding reactions with respect to protein reactions approach zero. The stationary solution is computed for the singularly-perturbed CME.

In order to illustrate the practical significance of our results, we work out several examples, some of which have not been studied before in the literature. As a first application, we discover that, with SPK, a self-regulating gene can exhibit bimodality even with non-cooperative binding to the promoter site. We then investigate the role of cooperativity. In contrast to deterministic systems, we find that cooperativity does not change the number of modes. Nevertheless, cooperativity adds extra degrees of freedom by allowing the network to tune the relative weight of each mode without changing its location.

As a second application, we revisit the classical toggle switch, under slow TF-gene binding/unbinding. It has been reported before that, with fast TF-gene binding/unbinding, the toggle switch with single-gene copies can be “bistable” without cooperative binding [42]. We show that this can also happen with SPK, and, moreover, that a new mode having both proteins at high copy numbers can emerge. We provide a method to calculate the weight of each mode and show that the third mode is suppressed for sufficiently high kinetic rates for the dimerization reactions.

A third application that we consider is a simplified model of synchronization of communicating toggle switches. In bacterial populations, quorum sensing has been proposed [43] as a way for bacterial cells to broadcast their internal states to other cells in order to facilitate synchronization. Quorum sensing communication has been adopted also as a tool in synthetic biology [44, 45]. Mathematical analysis of coupled toggle switches designs usually employs deterministic models [46]. We study a simplified stochastic model of coupled toggle switches with SPK and compare the resulting number of modes with deterministic equilibria.

Our final, and potentially most significant, application is motivated by cellular differentiation. A well-known metaphor for cell lineage specification arose from the 1957 work of Waddington [47], who imagined an “epigenetic landscape” with a series of branching valleys and ridges depicting stable cellular states. In that context, the emergence of new modes in cell fate circuits is often interpreted as the creation of new valleys in the epigenetic landscape, and (deterministic) multi-stability is employed to explain cellular differentiation [5]. However, an increasing number of studies have suggested stochastic heterogeneous gene expression as a mechanism for differentiation [13, 48, 49]. Numerical analysis of the CME for the canonical cell-fate circuit have shown the emergence of new modes due to SPK in such models [39, 50]. This general category of cell-fate circuits includes pairs such as PU.1:GATA1, Pax5:C/EBP*α* and GATA3:T-bet [51]. Cell fate circuits are characterized by TF cross-antagonism. However, their behavior is affected by the promoter configurations available for binding, the cooperativity index of the TFs, and the relative ratio of production rates. Hence, we study two models that differ in the aforementioned aspects and we highlight the differences between our findings and the behavior predicted by the corresponding deterministic model. The first model employs independent cooperative binding. We show that such a network can exhibit more than four modes. In contrast, the deterministic model predicts up to four modes only with cooperativity [52]. The second network is a PU.1/GATA.1 network which employs non-cooperative binding and a restricted set of promoter configurations. The deterministic model is monostable, while the parameters of the stochastic model can be chosen to have additional modes including the cases of bistability and tristability.

### The reaction network structure

In this paper, a GRN is formally defined as a set of nodes (genes) that are connected with each other through regulatory interactions via the proteins that the genes express. The regulatory proteins are called *transcription factors* (TFs). A TF regulates the expression of a gene by reversibly binding to the gene’s promoter and by either enhancing expression or repressing it.

The formalism we employ in order to describe GRNs at the elementary level is that of Chemical Reaction Networks (CRNs) [53]. A CRN consists of *species* and *reactions*, which we describe below.

#### Species

The species in our context consist of promoter configurations for the various genes participating in the network, together with the respective TFs expressed from these genes and some of their multimers. A configuration of a promoter is characterized by the possible locations and number of TFs bound to the promoter at a given time. If a promoter is expressed constitutively, then there are two configurations specifying the expression activity state, active or inactive. A multimer is a compound consisting of a protein binding to itself several times. For instance, dimers and trimers are 2-mers and 3-mers, respectively. If a protein forms an *n*^th^-order multimer then we say that it has a cooperativity index of *n*. If species is denoted by X, then its copy number is denoted by *X*. The set of all species is S.

For simplicity we assume the following:

(**A1**) Each promoter can have up to two TFs binding to it.;
(**A2**) Each TF is a single protein that has a fixed cooperativity index, i.e, it cannot act as a TF with two different cooperativity indices;
(**A3**) Each gene is present with only a *single copy*.

All the above assumptions can be relaxed. S1 Text §4,5 contain generalizations of the results to heterogeneous TFs, and arbitrary numbers of gene copy numbers.

Consider the *i*^th^ promoter. The expression rate of a gene is dependent on the current configuration of its promoter. We call the set of all possible such configurations the *binding-site set* *B*_*i*_. Each member of *B*_*i*_ corresponds to a configuration that translates into a specific species ${\text{D}}_{j}^{i},j\in B_{i}$. If a promoter has just one or no regulatory binding sites, then we let *B*_*i*_ = {0, 1}. Hence, the promoter configuration can be represented by *two species*: the unbound species ${\text{D}}_{0}^{i}$ and the bound species ${\text{D}}_{1}^{i}$. If the promoter has no binding sites then the promotor configuration species are interpreted as the inactive and active configurations, respectively. On the other hand, if the promoter has *two binding* sites then *B*_*i*_ = {00, 01, 10, 11} (We interpret the elements of the binding set as integers in binary representation). The first digit in a member of *B*_*i*_ specifies whether the first binding site is occupied, and the second digit specifies the occupancy of the second binding site. Hence, the promoter configuration can be represented by four species *${\text{D}}_{00}^{i},{\text{D}}_{10}^{i},{\text{D}}_{01}^{i},{\text{D}}_{11}^{i}$*. Note that in general we need to define 2^*κ*^ species for a promoter with *κ* binding sites.

The species that denotes the protein produced by the *i*^th^ gene is X_*i*_. A protein’s multimer is denoted by X_*ic*_. If protein X_*i*_ does not form a multimer then X_*ic*_ ≔ X_*i*_.

#### Reactions

**Fig 2:**
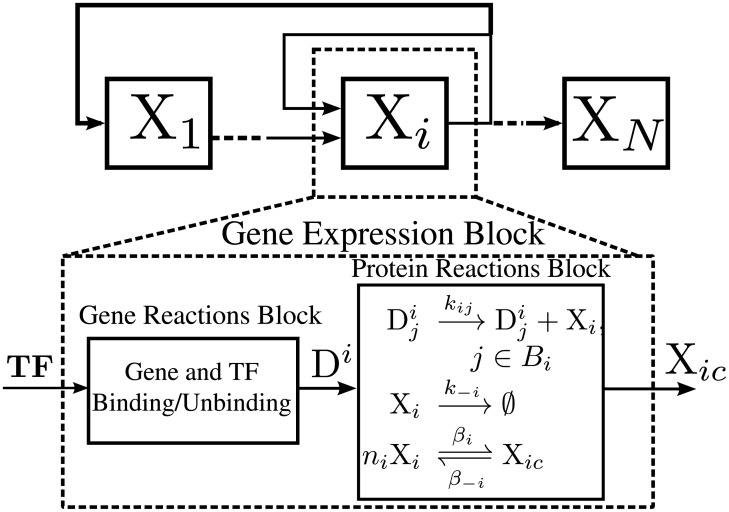
A gene expression block. A GRN that consists of gene expression blocks. A block consists of a gene reactions block and a protein reactions block. The gene reactions are described in the text. **TF** is a vector of TFs which can be monomers, dimers, or higher order multimers. D^*i*^ is a vector whose components consist of the ${\text{D}}_{j}^{i}$’s. The dimension of **TF** is equal to the number of binding sites of the gene.

In our context, the reactions consist of TFs binding and unbinding with promoters and the respective protein expression (with transcription and translation combined in one step), decay, and *n*-merization. For each gene, we define a *gene expression block*. Each block consists of a set of *gene reactions* and a set of *protein reactions* as shown in Fig 2.

If the promoter is constitutive, i.e. it switches between two configurations autonomously without an explicitly modeled TF-promoter binding, then *B*_*i*_ = {0, 1} and the gene reactions block consists of:

$$\begin{array}{c} {\text{D}}_{0}^{i}\overset{\alpha_{i}}{\underset{\alpha_{-i}}{⇌}}{\text{D}}_{1}^{i}. \end{array}$$

We refer to ${\text{D}}_{0}^{i}$ and $D_{1}^{i}$ as the *inactive* and *active* configurations, respectively. If the promoter has one binding site, then also *B*_*i*_ = {0, 1} and the gene reactions block consists of just two reactions:

$$\begin{array}{c} \text{TF}+{\text{D}}_{0}^{i}\overset{\alpha_{i}}{\underset{\alpha_{-i}}{⇌}}{\text{D}}_{1}^{i}, \end{array}$$

where ${\text{D}}_{0}^{i}$ and $D_{1}^{i}$ are the promoter configurations when *unbound* and *bound* to the TF, respectively. Note that we did not designate a specific species as the active one since it depends on whether the TF is an activator or a repressor. Specifically, when TF is an activator, $D_{1}^{i}$ will be the active configuration and $D_{0}^{i}$ will be the inactive configuration, and vice versa when TF is a repressor. Finally, if the promoter has two TFs binding to it, then they can bind *independently*, *competitively*, or *cooperatively*. Cooperative binding is discussed in S1 Text §4.3.1. If they bind independently, then the promoter has two binding sites. Hence, *B*_*i*_ = {00, 01, 10, 11} and the gene block contains the following *gene reactions*:

$$\begin{array}{c} {\text{TF}}_{1}+{\text{D}}_{00}^{i}\overset{\alpha_{i1}}{\underset{\alpha_{-i1}}{⇌}}{\text{D}}_{10}^{i}\\ {\text{TF}}_{1}+{\text{D}}_{01}^{i}\overset{\alpha_{i2}}{\underset{\alpha_{-i2}}{⇌}}{\text{D}}_{11}^{i},\\ {\text{TF}}_{2}+{\text{D}}_{00}^{i}\overset{\alpha_{i3}}{\underset{\alpha_{-i3}}{⇌}}{\text{D}}_{01}^{i},\\ {\text{TF}}_{2}+{\text{D}}_{10}^{i}\overset{\alpha_{i4}}{\underset{\alpha_{-i4}}{⇌}}{\text{D}}_{11}^{i}. \end{array}$$

The activity of each configuration species is dependent on whether the TFs are activators or repressors, and on how they behave jointly. This can be characterized fully by assigning a production rate for each configuration as will be explained below. In the case of competitive binding, two different TFs compete to bind to the same location. This can be modeled similarly to the previous case except that the transitions to ${\text{D}}_{11}^{i}$, i.e. the configuration where both TFs are bound, are not allowed. Hence, the gene reactions block will have only the first and third, and the binding set reduces to *B*_*i*_ = {00, 01, 10}.

Our binding/unbinding reaction models account for the stoichiometric change in proteins when binding to the promoters, and the bound proteins are accounted for by designating a gene state for each promoter configuration (defined by the number and location of bound proteins). The bound protein molecules are only governed by slow gene reactions until they are released.

We assume that RNA polymerase and ribosomes are available in high copy numbers, and that we can lump transcription and translation into one simplified “production” reaction. The latter assumption is a reasonable approximation since the turnover of mRNA is typically faster than that of protein. The rate of production is dependent on the promoter’s configuration. So for each configuration ${\text{D}}_{j}^{i},j\in B_{i}$ the *production* reaction is:

$$\begin{array}{c} {\text{D}}_{j}^{i}\overset{k_{ij}}{\to }{\text{D}}_{j}^{i}+{\text{X}}_{i}, \end{array}$$

where the kinetic constant *k*_*ij*_ is a non-negative number. The case *k*_*ij*_ = 0 means that when the promoter configuration is ${\text{D}}_{j}^{i}$ there is no protein production, and hence $D_{j}^{i}$ is an inactive configuration. The promoter configuration can be ranked from the most active to the least active by ranking the corresponding production kinetic rate constants.

Consequently, the character of a TF is manifested as follows: if the maximal protein production occurs at a configuration with the TF being bound we say that the TF is *activating*, and if the reverse holds it is *repressing*. And, if the production is maximal with multiple configurations such that the TF is bound in some of them and unbound in others then the TF is neither repressing nor activating.

We model decay and/or dilution as a single reaction:

$$\begin{array}{c} {\text{X}}_{i}\overset{k_{-i}}{\to }\emptyset . \end{array}$$

The expressed proteins can act as TFs. They may combine to form dimers or higher order multimers before acting as TFs. The numbers of copies of the TF needed to form a multi-mer is called the *cooperativity index* and we denote it by *n*. Hence, we model the cooperativity reactions as given in Fig 2 as follows (called the *n-imerization* reactions):

$$\begin{array}{c} n{\text{X}}_{i}\overset{\beta_{i}}{\underset{\beta_{-i}}{⇌}}{\text{X}}_{ic}. \end{array}$$

If the cooperativity index of X_*i*_ is 1, then the species X_*ic*_ ≔ X_*i*_, and the multimerization reaction becomes empty. Higher order multi-merization processes can be modelled as multi-step or sequential reactions [54]. We discuss how our theory includes this case in S1 Text §4.3.3, by showing how an equivalent one-step model can be formulated.

#### Kinetics

In order to keep track of molecule counts, each species $Z_{i}\in S$ is associated with a copy number $z_{i}\in Z_{\geq 0}$. To each reaction R_*j*_ one associates a propensity function *R*_*j*_. We use the well-known *Mass-Action Kinetics*, which are reviewed in S1 Text §1.1.

#### A gene regulatory network

Consider a set of *N* genes, binding sets ${\left\{B_{i}\right\}}_{i=1}^{N}$, and kinetic constants *k*_*j*_’s. A *gene expression block*, as shown in Fig 2, is a set of gene reactions and protein reactions as defined above. Each gene block has an output that is either the protein or its *n*-mer, and it is designated by X_*ic*_. The input to each gene expression block is a subset of the set of the outputs of all blocks. Then, a GRN is an arbitrary interconnection of gene expression blocks (Fig 2). S1 Text §4 defines a more general class of network that we can study. A directed graph can be associated with a GRN as follows. Each vertex corresponds to a gene expression block. There is a directed edge from vertex A to vertex B if the output of A is an input to B. For simplicity, we assume the following: (**A4**) The graph of gene expression blocks is connected. Note that if A4 is violated, our analysis can be applied to each connected component.

#### Time-scale separation

As mentioned in the introduction, we assume that the gene reactions are considerably slower than the protein reactions. In order to model this assumption, we write the kinetic rates of gene reactions in the form *εk*_*j*_, where 0 < *ε* ≪ 1 and assume that all other kinetic rates (for protein production, decay and multi-merization) are *ε*^−1^-times faster.

Events in biological cells usually take place at different time-scales [1], and hence singular perturbation techniques are widely used in deterministic settings in order to reduce models for analysis. On the other hand, model-order reduction by time-scale separation in stochastic processes has been mainly used in the literature for computational purposes, for example to accelerate the stochastic simulation algorithm [55, 56], or to compute finite-space-projection solutions to the CME [57]. In this work, we use a singular perturbation approach for the analytical purpose of characterizing the form of the stationary PMF in the regimes of slow gene-TF binding/unbinding.

In the case of a finite MC, the CME is a finite-dimensional linear ODE, and reduction methods for linear systems can be used [41] and applied to MCs [58, 59]. For continuous-time MCs on a countable space, as needed when analyzing gene networks, there are difficult and open technical issues. Exponential stochastic stability [60] needs to be established for the stationary solution in order to guarantee the existence of the asymptotic expansion in *ε* [61]. Although it has been shown for a class of networks [62], the general problem needs further research. In this paper, we will not delve into technical issues of stochastic stability; we assume that these expansions exist and that the solutions converge to a unique equilibrium solution.

### Dynamics and the CME

The dynamics of the network refers to the manner in which the *state* evolves in time, where the state $Z\left(t\right)\in Z\subset Z_{\geq 0}^{\left|S\right|}$ is the vector of copy numbers of the species of the network at time *t*. The standard stochastic model for a CRN is that of a continuous-time MC. Let the state be *Z*(*t*) = *z* ∈ **Z**, where **Z** is the state-space. The relevant background is reviewed in S1 Text §1.1.

Let *p*_*z*_(*t*) = Pr[*Z*(*t*) = *z*|*Z*(0) = *z*_0_] be the stationary PMF for any given initial condition *z*_0_. Its time evolution is given by the CME.

Since our species are either gene species or protein species, we split the stochastic process *Z*(*t*) into two subprocesses: *the gene process* *D*(*t*) and *the protein process* *X*(*t*), as explained below.

For each gene we define one process *D*_*i*_ such that *D*_*i*_(*t*) ∈ *B*_*i*_. *D*_*i*_(*t*) = *j* if and only the promoter configuration is encoded by *j* ∈ *B*_*i*_. Collecting these into a vector, define the gene process *D*(*t*) ≔ [*D*_1_(*t*), …, *D*_*N*_(*t*)]^*T*^ where $D\left(t\right)\in \prod_{i=1}^{N}B_{i}$. The *i*^th^ gene can be represented by |*B*_*i*_| states, so $L≔\prod_{i=1}^{N}\left|B_{i}\right|$ is the total number of promoter configurations in the GRN. With abuse of notation, we write also *D*(*t*) ∈ {0, ‥, *L* − 1} in the sense of the bijection between {0, ‥, *L* − 1} and $\prod_{i=1}^{N}B_{i}$ defined by interpreting *D*_1_…*D*_*N*_ as a binary representation of an integer. Hence, *d* ∈ {0, ‥, *L* − 1} corresponds to (*d*_1_, …, *d*_*N*_) ∈ *B*_1_ × ‥ × *B*_*N*_ and we write *d* = (*d*_1_, ‥, *d*_*N*_).

Since each gene expresses a corresponding protein, we define $X_{i1}\left(t\right)\in Z_{\geq 0},i=1,‥,N$ protein processes. If the multimerized version of the *i*^th^ protein participates in the network as an activator or repressor then we define *X*_*ic*_(*t*) as the corresponding multimerized protein process, and we denote *X*_*i*_(*t*) ≔ [*X*_*i*1_(*t*), *X*_*ic*_(*t*)]^*T*^. If there is no multimerization reaction then we define *X*_*i*_(*t*) ≔ *X*_*i*1_(*t*). Since not all proteins are necessarily multimerized, the total number of protein processes is *N* ≤ *M* ≤ 2*N*. Hence, the *protein process* is $X\left(t\right)={\left[X_{1}^{T}\left(t\right),‥,X_{N}^{T}\left(t\right)\right]}^{T}\in Z_{\geq 0}^{M}$ and $Z=Z_{\geq 0}^{M}\times \prod_{i=1}^{N}B_{i}$.

## Results

### Decomposition of the CME

It is crucial to our analysis to represent the linear system of differential equations given by the CME as an interconnection of weakly coupled linear systems. To this end, we present the appropriate notation in this subsection.

Consider the joint PMF: *p*_*d*,*x*_(*t*) = Pr[*X*(*t*) = *x*, *D*(*t*) = *d*], which represents the probability at time *t* that the protein process *X* takes the value $x\in Z_{+}^{M}$ and the gene process *D* takes the value *d* ∈ {0, ‥, *L* − 1}. Recall that *x* is a vector of copy numbers for the protein processes while *d* encodes the configuration of each promoter in the network. Then, we can define for each fixed *d*: $p_{d}\left(t\right)≔{\left[p_{dx_{0}}\left(t\right),p_{dx_{1}}\left(t\right),\ldots .\right]}^{T}$, representing the vector enumerating the joint probabilities for all values of *x* and for a fixed *d*, where *x*_0_, *x*_1_, ‥ is an indexing of $Z_{\geq 0}^{M}$. Note that *p*_*d*_(*t*) can be thought of as an infinite vector with respect to the aforementioned indexing. Finally, let

$$\begin{array}{c} p\left(t\right)≔{\left[p_{0}{\left(t\right)}^{T},\ldots ,p_{L-1}^{T}\left(t\right)\right]}^{T}, \end{array}$$ (1)

representing a concatenation of the vectors *p*_*d*,*x*_(*t*) for *d* = 0, ‥, *L* − 1. Note that *p*(*t*) is a finite concatenation of infinite vectors. The joint stationary PMF $\bar{\pi }$ is defined as the following limit, which we assume to exist and is independent of the initial PMF: $\bar{\pi }={\text{lim}}_{t\to \infty }p\left(t\right)$. Note that $\bar{\pi }$ depends on *ε*.

Consider a given GRN. The CME is defined over a countable state space **Z**. Hence, the CME can be interpreted as an infinite system of differential equations with an infinite infinitesimal generator matrix Λ which contains the reaction rates (see S1 Text §1.1).

Consider partitioning the PMF vector as in (1). Recall that reactions have been divided into two sets: slow gene reactions and fast protein reactions. This allows us to write Λ as a sum of a slow matrix $\varepsilon \hat{\Lambda }$ and a fast matrix $\tilde{\Lambda }$, which we call a fast-slow decomposition. Furthermore, $\tilde{\Lambda }$ can be written as a block diagonal matrix with *L* diagonal blocks which correspond to conditioning the MC on a specific gene state *d*. This is stated in the following basic proposition (see S1 Text §2.1 for the proof):

**Proposition 1**. *Given a GRN*. *Its CME can be written as*

$$\begin{array}{c} \dot{p}\left(t\right)=\Lambda_{\varepsilon }p\left(t\right)=(\tilde{\Lambda }+\varepsilon \hat{\Lambda })p\left(t\right), \end{array}$$ (2)

*where* $p\left(t\right)={\left[p_{0}^{T}\left(t\right),‥,p_{L-1}^{T}\left(t\right)\right]}^{T}$, *and*

$$\begin{array}{c} \tilde{\Lambda }=diag\left[\Lambda_{0},‥,\Lambda_{L-1}\right] \end{array}$$ (3)

*where* $\tilde{\Lambda }$ *is the fast matrix*, $\hat{\Lambda }$ *is the slow matrix*, *and* Λ_0_, ‥, Λ_*L*−1_ *are stochastic matrices*.

### Conditional MCs

For each *d*, consider modifying the MC *Z*(*t*) defined in the previous section by replacing the stochastic process *D*(*t*) by a deterministic constant process *D*(*t*) = *d*. This means that the resulting MC does not describe the gene process dynamics, it only describes the protein process dynamics *conditioned on* *d*. Henceforth, we refer to the resulting MC as the *MC conditioned on d*. The infinitesimal generator of a MC conditioned on *d* is denoted by Λ_*d*_, and is identical to the corresponding block on the diagonal of $\tilde{\Lambda }$ as given in (3). In other words, fixing *D*(*t*) = *d* ∈ {0, ‥, *L* − 1}, the dynamics of the network can be described by a CME:

$${\dot{p}}_{X|d}=\Lambda_{d}p_{X|d},$$ (4)

where *p*_*X*|*d*_ is a vector that enumerates the conditional probabilities *p*_*x*|*d*_ = Pr[*X*(*t*) = *x*|*D*(*t*) = *d*] for a given *d*. The conditional stationary PMF is denoted by: $\pi_{X|d}^{\left(J\right)}={\text{lim}}_{t\to \infty }p_{X|d}\left(t\right)$, where (*J*) refers to the fact that it is joint in the protein and multimerized protein processes. Note that $\pi_{X|d}^{\left(J\right)}$ is independent of *ε*. This notion of a conditional MC is useful since, at the SPK limit, *D*(*t*) stays constant. It can be noted from (3) that when *ε* = 0 the dynamics of *p*_*d*_ decouples and becomes independent of $p_{\tilde{d}},\tilde{d}=0,‥,L-1,\tilde{d}\neq d$.

We show below that each conditional MC has a simple structure. Fixing the promoter configuration *D*(*t*) = *d* = (*d*_1_, ‥, *d*_*N*_), the network consists of *uncoupled* birth-death processes. So for each *d*_*i*_, the protein reactions of production and dimerization corresponding to the *i*^th^ promoter can be written as follows without multimerization: $\emptyset \overset{k_{id_{i}}}{\underset{k_{-i}}{⇌}}{\text{X}}_{i}$, where the subscript *id*_*i*_ refers to the production kinetic constant corresponding to the configuration species ${\text{D}}_{d_{i}}^{i}$, or, if there is a multimerization reaction, it takes the form: $\emptyset \overset{k_{id_{i}}}{\underset{k_{-i}}{⇌}}{\text{X}}_{i},\;n_{i}{\text{X}}_{i}\overset{\beta_{i}}{\underset{\beta_{-i}}{⇌}}{\text{X}}_{ic}$. Note that the stochastic processes X_*i*_(*t*), *i* = 1, ‥, *N* conditioned on *D*(*t*) = *d* are independent of each other. Hence, the conditional stationary PMF $\pi_{X|d}^{\left(J\right)}$ can be written as a product of stationary PMFs and the individual stationary PMFs have Poisson expressions. The following proposition gives the analytic expression of the conditional stationary PMFs: (see S1 Text §2.2 for proof).

**Proposition 2**. *Fix d* ∈ {0, ‥, *L* − 1}. *Consider* (4), *then there exists a conditional stationary PMF* $\pi_{X|d}^{\left(J\right)}$ *and it is given by*

$$\begin{array}{c} \pi_{X|d}^{\left(J\right)}\left(x\right)=\prod_{i=1}^{N}\pi_{X|di}\left(x_{i}\right), \end{array}$$ (5)

*where*

$$\begin{array}{c} \pi_{X|d_{i}}^{\left(J\right)}\left(x_{i}\right)=\{\begin{array}{cc} P(x_{i1},x_{i2};\frac{k_{id_{i}}}{k_{-i}},\frac{k_{id_{i}}^{n_{i}}\beta_{i}}{n_{i}!k_{-i}^{n_{i}}\beta_{-i}}) & \;\;\;\;\;:n_{i}>1\\ P(x_{i};\frac{k_{id_{i}}}{k_{-i}}) & \;\;\;\;\;:n_{i}=1, \end{array} \end{array}$$ (6)

*where* (*J*) *refers to the joint PMF in multimerized and non-multimerized processes*, *x*_*i*1_ *refers to the copy number of* X_*i*_, *while x*_*i*2_ *refers to the copy number of* X_*ic*_, $P\left(x;a\right)≔\frac{a^{x}}{x!}e^{-a},P\left(x_{1},x_{2};a_{1},a_{2}\right)≔\frac{a_{1}^{x_{1}}}{x_{1}!}\frac{a_{2}^{x_{2}}}{x_{2}!}e^{-a_{1}-a_{2}}$.

**Remark 1**. *The conditional PMF in* (5) *is a joint PMF in the protein and multimerized protein processes*. *If we want to compute a marginal stationary PMF for the protein process only*, *then we average over the multimerized protein processes X*_*ic*_, *i* = 1, ‥, *N* *to get a joint Poisson in N variables*. *Hence*, *the formulae* (5)-(6) *can be replaced by*:

$$\begin{array}{c} \pi_{X|d}\left(x\right)≔\sum_{i=1}^{M-N}\sum_{x_{i2}=0}^{\infty }\pi_{X|d}^{\left(J\right)}\left(x\right)=\prod_{i=1}^{N}P(x_{i};\frac{k_{id_{i}}}{k_{-i}}), \end{array}$$ (7)

*where M* − *N is the number of n-merized protein processes, and π*_*X*|*d*_ *is the marginal stationary PMF for the protein process*.

### Decomposition of the stationary distribution

Recall the slow-fast decomposition of the CME in (2) and the joint stationary PMF $\bar{\pi }$. In order to emphasize the dependence on *ε* we denote ${\bar{\pi }}^{\varepsilon }≔\bar{\pi }\left(\varepsilon \right)$. Hence, ${\bar{\pi }}^{\varepsilon }$ is the unique stationary PMF that satisfies $\Lambda_{\varepsilon }{\bar{\pi }}^{\varepsilon }=0$, *π*^*ε*^ > 0, and $\sum_{z}\pi_{z}^{\varepsilon }=1$, where the subscript denotes the value of the stationary PMF at *z*.

Our aim is to characterize the stationary PMF as *ε* → 0. Writing ${\bar{\pi }}_{\varepsilon }$ as an asymptotic expansion to first order in terms of *ε*, we have

$$\begin{array}{c} {\bar{\pi }}^{\varepsilon }={\bar{\pi }}^{\left(0\right)}+{\bar{\pi }}^{\left(1\right)}\varepsilon +o\left(\varepsilon \right). \end{array}$$ (8)

Our aim is to find ${\bar{\pi }}^{\left(0\right)}$. We use singular perturbations techniques to derive the following theorem (see S1 Text §2.3):

**Theorem 3**. *Consider a given GRN with L promoter states with the CME* (2). *Writing* (8), *then the joint stationary PMF* $\bar{\pi }≔{\text{lim}}_{\varepsilon \to 0^{+}}{\bar{\pi }}^{\varepsilon }$ *can be written as*: $\bar{\pi }\left(x,d\right)=\sum_{d=0}^{L-1}\lambda_{d}{\bar{\pi }}_{X|d}\left(x,d\right)$, *where* λ = [λ_0_, ‥, λ_*L*−1_]^*T*^ *is the principal normalized eigenvector of*:

$$\begin{array}{c} \Lambda_{r}≔diag[1^{T},‥,1^{T}]\hat{\Lambda }\;\left[{\bar{\pi }}_{X|0}\;{\bar{\pi }}_{X|1}\;\ldots \;{\bar{\pi }}_{X|L-1}\right], \end{array}$$ (9)

*where* ${\bar{\pi }}_{X|0},‥,{\bar{\pi }}_{X|L-1}$ *are the extended conditional stationary PMFs defined as*: ${\bar{\pi }}_{X|d}\left(x,d\right)=\pi_{X|d}\left(x\right)$, ${\bar{\pi }}_{X|d}\left(x,d^{\prime }\right)=0$ *when d*′ ≠ *d*.

The result characterizes the stationary solution of (2) which is a joint PMF in *X* and *D*. However, we are particularly interested in the marginal stationary PMF of the protein process *X* and the marginal stationary PMF of the non-multimerized protein process, since these PMFs are typically experimentally observable. Therefore, we can use Remark 1 to write the stationary PMF as mixture of *L* Poisson distributions with weights ${\left\{\lambda_{d}\right\}}_{d=0}^{L-1}$:

**Corollary 4**. *Consider a given GRN with L genes with the CME* (2). *Writing* (8), *let π*_*X*|0_, …, *π*_*X*|*L*−1_ *be the conditional stationary PMFs of* Λ_0_, …, Λ_*L*−1_, *where explicit expressions are given in* (5). *Then*, *we can write the following* $\pi^{\left(J\right)}(x)≔{\text{lim}}_{\varepsilon \to 0^{+}}{\text{lim}}_{t\to \infty }\text{Pr}[X(t)=x]=\sum_{d=0}^{L-1}\lambda_{d}\pi_{X|d}^{\left(J\right)}(x),$ *where* λ = [λ_0_, ‥, λ_*L*−1_]^*T*^ *is as given Theorem 3*. *Furthermore*, *the marginal stationary PMF of the non-multimerized protein process can be written as*:

$$\begin{array}{c} \pi \left(x\right)\;\;≔\;\;\sum_{d=0}^{L-1}\lambda_{d}\pi_{X|d}\left(x\right)\;\;=\;\;\sum_{d=0}^{L-1}\lambda_{d}\prod_{i=1}^{N}P(x_{i};\frac{k_{id_{i}}}{k_{-i}}). \end{array}$$ (10)

**Remark 2**. *In the remainder of the Results section, when we refer to the* “*stationary PMF*” *we mean the marginal stationary PMF of the non-multimerized protein process given in* (10).

**Remark 3**. *If a mode is defined as a local maximum of a stationary PMF*, *then this does not necessarily imply that the stationary PMF has L modes since the peak values of two Poisson distributions can be very close to each other*. *In the remainder of the paper we will call each Poisson distribution in the mixture as a* “*mode*” *in the sense that it represents a component in the mixture PMF*. *The number of local maxima of a PMF can be found easily given the expression* (10).

### The reduced-order finite MC

The computation of the weighting vector λ in Theorem 3 requires computing the *L* × *L* matrix Λ_*r*_ in (9) which can be interpreted as the infinitesimal generator of an *L*-dimensional MC. The expression in (9) involves evaluating the product of infinite dimensional matrices. Since the structure of the GRN and the form of the conditional PMF in (5) are known, an easier algorithm to compute Λ_*r*_ for our GRNs is given in Proposition SI-2 in S1 Text. The algorithm provides an intuitive way to interpret Theorem 3 and can be informally described as follows.

Let *D*(*t*) = *d*, the algorithm implies that each binding reaction of the form: $\text{TF}+{\text{D}}_{d_{i}}^{i}\overset{\alpha }{\to }{\text{D}}_{d_{i^{\prime }}}^{i}$, gives the rate αE[TF|D=d], where E denotes mathematical expectation. Hence it corresponds to a reaction of the following form in the reduced-order MC:

$$\begin{array}{c} {\text{D}}_{d_{i}}^{i}\overset{\alpha E[\text{TF}|D=d]}{\to }{\text{D}}_{d_{i^{\prime }}}^{i}. \end{array}$$ (11)

Using Proposition 2, we can write: (See S1 Text §2.4)

$$\begin{array}{c} E\left[\text{TF}|D=d\right]=\frac{\alpha }{n_{i}!}\frac{\beta_{i}}{\beta_{-i}}(\frac{k_{id_{i}}}{k_{-i}}{)}^{n_{i}}. \end{array}$$ (12)

### Generalization

Theorem 3 and Corollary 4 have been stated for GRNs that have gene expression blocks of the form given in Fig 2. Nevertheless, the same results can also be stated for a larger class of networks. The generalized class consists of GRNs with weakly reversible deficiency zero conditional Markov chains. The stationary PMF for networks in this class can also be expressed as a mixture of Poisson PMFs. This enables us to include networks with hetero-dimerization, diffusion and multi-step multi-merization in our study. The full details are in S1 Text §4, and a diffusion-based interconnection of toggle switches will be studied as an example.

### The gene bursting model

The simplest network is the unregulated gene which is used for transcriptional bursting [17] and studied using time-scale separation in [32, 63]. Consider:

$$\begin{array}{c} {\text{D}}_{0}\overset{\varepsilon \alpha }{\underset{\varepsilon \alpha_{-}}{⇌}}{\text{D}}_{1}\\ {\text{D}}_{1}\overset{k}{\to }\text{X}+{\text{D}}_{1},\\ \;\text{X}\overset{k_{-}}{\to }0. \end{array}$$ (13)

Referring to Fig 2, we identify a single gene block with two states. Using (5), the conditional stationary PMFs are two Poissons at 0 and *k*/*k*_−_, and the stationary PMF is a bimodal mixture of them with weights *α*_−_/(*α* + *α*_−_), *α*/(*α* + *α*_−_), respectively (See S1 Text §3.1). In the case of fast promoter kinetics, the resulting stationary PMF is a Poisson with mean $\frac{\alpha }{\alpha +\alpha_{-}}\frac{k}{k_{-}}$ which coincides with the deterministic equilibrium. Although both stochastic models share the mean, the stationary PMFs and their variances differ drastically.

**Fig 3:**
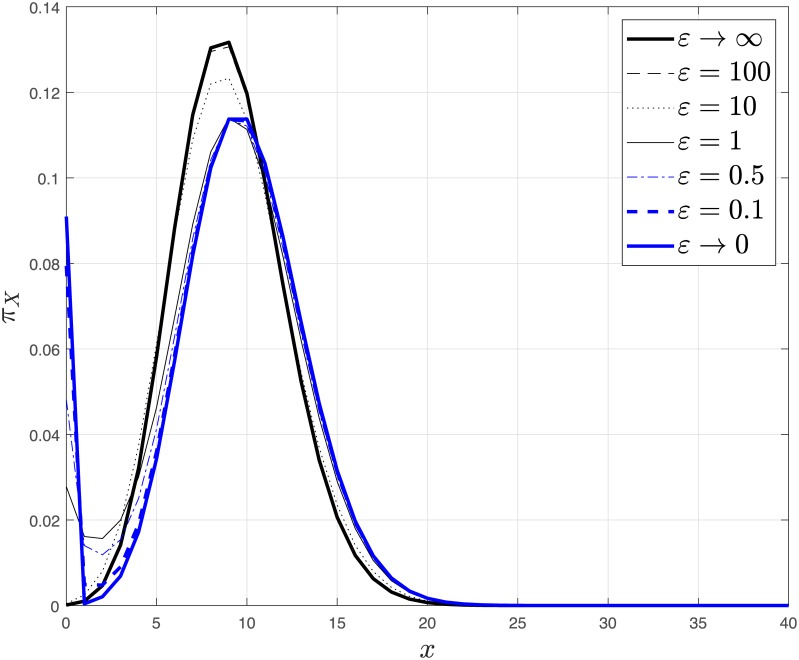
The stationary probability distribution for different *ε* which shows the transition from fast promoter kinetics, i.e., *ε* → ∞, to slow promoter kinetics, i.e., *ε* → 0, in a single unregulated gene. The stationary distribution is bimodal for small *ε*, i.e. *ε* ≤ 1, and unimodal for large *ε*. The deterministic equilibrium coincides with the fast kinetics mode at $\frac{\alpha }{\alpha +\alpha_{-}}\frac{k}{k_{-}}$. The slow kinetic limit is calculated via Corollary 4, the fast kinetics limit is a Poisson centered at the deterministic equilibrium, while the remaining curves are computed by evaluating the exact solution given in [64]. The parameters are *α*_−_ = 0.1, *α* = 1, *k*_−_ = 2, *k* = 20.

Fig 3 shows the transition from fast to slow promoter kinetics using the exact solution [64] and compares it to the predicted mixture of two Poissons.

### The role of cooperativity

A TF is said to be cooperative if it acts only after it forms a dimer or a higher-order *n*-mer that binds to the gene’s promoter [54]. In standard deterministic modelling, a cooperative activation changes the form of the quasi-steady state activation rate from a Michaelis-Menten function into a Hill function. Cooperativity is often necessary for a network to have multiple equilibria in some kinetic parameter ranges. For example, a non-cooperative self-activating gene can only be mono-stable, while its cooperative counterpart can be multi-stable for some parameters.

Corollary 4 and (12) show that cooperativity plays in the context of SPK a role that is very different from the deterministic setting. This is since the stationary PMF is a mixture of *L* Poisson processes (7) which are independent of the TFs’ cooperativity indices and ratios. In the non-cooperative case, a certain mode can be made more probable only by changing either the location of the mode or the dissociation ratio (the ratio of the binding to unbinding kinetic constants). On the other hand, a multimerized TF gives extra tuning parameters, namely the multimerization ratio and the cooperativity index. Hence, a certain mode can be made more or less probable by modifying either of them without changing the location of the peaks or the dissociation ratio. In order to illustrate the above idea, we analyze a self-regulating gene with SPK with and without cooperativity.

### A self-regulating gene

Consider a non-cooperative self-regulating gene. The unbound and bound gene states are *D*_0_, *D*_1_ with *k*_0_, *k*_1_ production rates, respectively. The network is activating if *k*_1_ > *k*_0_, and repressing otherwise.

Similar to the previous example, the stationary PMF is a mixture of two Poissons centered at *k*_0_/*k*_−_, *k*_1_/*k*_−_ with weights *α*_−_/(*αρ* + *α*_−_), *αρ*/(*αρ* + *α*_−_), respectively, where $\rho =E\left[X|D=0\right]=k_{0}/k_{-}.$ (Refer to (12) and S1 Text §3.2).

Next, consider the cooperative counterpart with dimerization rates *β*, *β*_−_. The stationary PMF stays the same except for $\rho =E\left[X_{2}|D=0\right]=k_{0}^{2}\beta /(2k_{-}^{2}\beta_{-}).$ Hence, in both cases, the PMF has modes at *k*_1_/*k*_−_, *k*_0_/*k*_−_, where the weight of the first mode is proportional to *ρ* which can be used in order to tune the weights freely while keeping the modes and the dissociation ratio unchanged. For instance, the PMF can be made effectively unimodal with a sufficiently high *ρ*.

#### Comparison with the deterministic model

**Fig 4:**
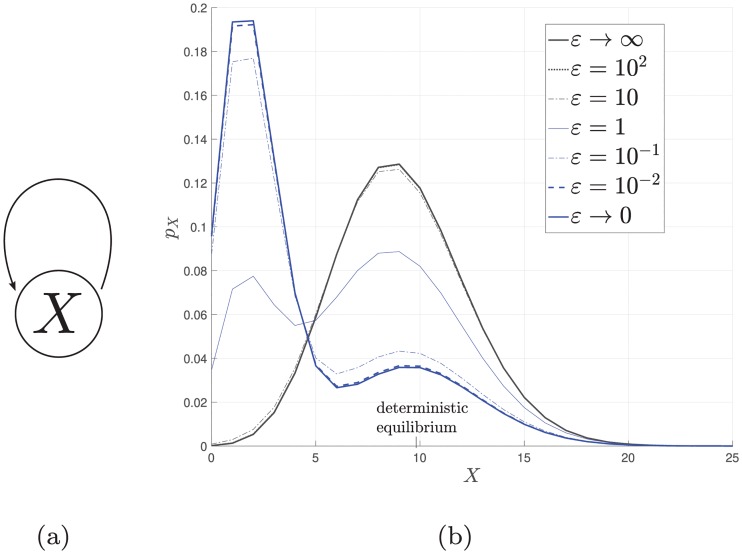
**More modes emerge due to SPK in cooperative self-activating gene** (a) A self-activating gene. (b) The stationary PMF for different *ε* which shows the transition from fast promoter kinetics to SPK in a leaky cooperative self-activation of a gene with cooperativity index 2. The slow kinetic limit is calculated via (10), while the remaining curves are computed by a finite projection solution [57] of the CME. The parameters are *α* = *α*_ = *ε*, *k*_0_ = 20, *k*_1_ = 100, *k*_ = 10, *β* = 10, *β*_ = 50.]

Table 1 compares the number of stable equilibria in the deterministic model with the number of modes in the stochastic model in the case of a single gene copy. There is no apparent correlation between the numbers of deterministic equilibria and stochastic modes. Fig 4 depicts the transition from a unique mode with fast promoter kinetics to multiple modes with SPK with a cooperative leaky self-activating gene.

**Table 1:** Comparing the number of stable equilibria/modes for a self-activating gene between stochastic with SPK and deterministic modelling frameworks. Details are given in S1 Text §6.3, where leakiness means that the unbound state has a non-zero expression rate.

	Non-Cooperative		Cooperative	
	Leaky	Non-Leaky	Leaky	Non-Leaky
Stochastic (Slow Promoter Kinetics)	2	1 (at 0)	2	1 (at 0)
Deterministic	1	1	1-2	1-2

### The toggle switch

**Fig 5:**
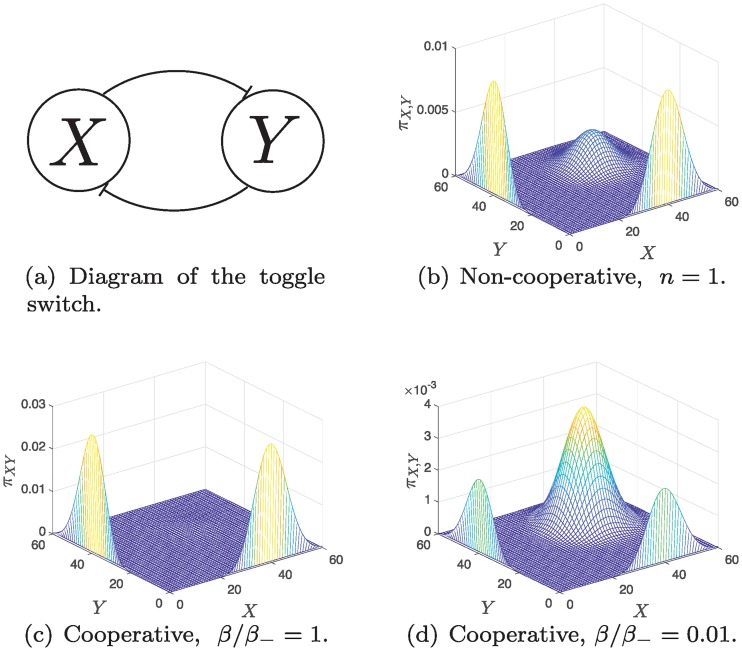
Cooperativity enables tuning of modes’ weights. Comparison of the stationary PMF between non-cooperative and cooperative binding. For all cases: *α*/*α*_ = 1/200, *k*_0_/*k*_ = 40. (a) Diagram of the toggle switch. (b) The stationary PMF for the non-cooperative case. (c) The stationary PMF for the cooperative case with *n* = 2, *β*/*β*_ = 1. (d) The stationary PMF for the cooperative case *β*/*β*_ = 0.01. All surfaces are plotted using (10).

A toggle switch is a basic GRN that exhibits deterministic multi-stability. It has two stable steady states and can switch between them with an external input or via noise. The basic design is a pair of two mutually repressing genes as in Fig 5a. The ideal behavior is that only one gene is “on” at any moment in time. The network consists of two *identical* genes whose expressed proteins X, Y act as TFs for each other (The general toggle switch is discussed in S1 Text §3.3). Each gene has dissociation ratio *α*/*α*_, production ratio *k*_0_/*k*_ for the unbound state only, multimerization ratio *β*/*β*_, and cooperativity index *n*. Using the algorithm given in Proposition SI-2 in S1 Text, the reduced-order Markov chain infinitesimal generator is:

$$\begin{array}{c} \Lambda_{r}=[\begin{array}{cccc} -\alpha \rho -\alpha \rho & \alpha_{-} & \alpha_{-} & 0\\ \alpha \rho & -\alpha_{-} & 0 & \alpha_{-}\\ \alpha \rho_{2} & 0 & -\alpha_{-} & \alpha_{-}\\ 0 & 0 & 0 & -\alpha_{-}-\alpha_{-} \end{array}], \end{array}$$ (14)

where $\rho =\frac{k_{0}^{n}}{k_{-}^{n}}\frac{\beta }{n!\beta_{-}}$. Notice immediately that the transition rates towards the configuration (1,1) are zero, which implies that the weight of the mode corresponding to (1, 1) is zero. Hence, we have three modes only. The weights corresponding to the modes can be found as the principal eigenvector of Λ_*r*_ as given in Corollary 4. Hence, the stationary distribution for *X*, *Y* is:

$$\begin{array}{c} \pi \left(x,y\right)=\frac{1}{2\frac{\alpha }{\alpha_{-}}\rho +1}(P\left(y;\frac{k_{0}}{k_{-}}\right)P\left(x;\frac{k_{0}}{k_{-}}\right)+\frac{\alpha }{\alpha_{-}}\rho P\left(y;\frac{k_{0}}{k_{-}}\right)\delta \left(x\right)+\frac{\alpha }{\alpha_{-}}\rho P\left(x;\frac{k_{0}}{k_{-}}\right)\delta \left(y\right)). \end{array}$$

Hence, we get that the PMF has three modes only at $\left(0,\frac{k_{0}}{k_{-}}\right)$,$\left(\frac{k_{0}}{k_{-}},0\right)$, $\frac{k_{0}}{k_{-}},\left(\frac{k_{0}}{k_{-}}\right)$ with relative weights $\frac{\alpha }{\alpha_{-}}\rho$,$\frac{\alpha }{\alpha_{-}}\rho$, 1, respectively. Since the stationary PMF has three modes, it deviates from the ideal behavior of a switch where at most two stable steady states, under appropriate parameter conditions, are possible. Nevertheless, a bimodal PMF can be achieved by minimizing the weight of the first mode at $\left(\frac{k_{0}}{k_{-}},\frac{k_{0}}{k_{-}}\right)$. If we fix *α*/*α*_, then this can be satisfied by tuning *n*, *β*/*β*_ to maximize *ρ*. Choosing higher cooperativity indices, subject to *n* < *k*_0_/*k*_, achieves this also. For instance, a standard asymmetric design [65] uses cooperativity indices 2, 3. Fig 5 depicts the effect of cooperativity on achieving the desired behavior with the same dissociation constant and production ratios. Notice that cooperativity allows us to minimize or maximize the weight of the (high,high) mode by tuning the dimerization ratio.

The toggle switch has three modes regardless of the cooperativity index. This is unlike the deterministic model where only one positive stable state is realizable with non-cooperative binding, and two stable steady states are realizable with cooperative binding. S1 Text §3.3 contains further Monte-Carlo simulations that show that the predicted third mode appears with a two-to-one time scale separation between the slow gene reactions and the fast protein reactions. Experimentally, a recent study has reported that the CRI-Cro toggle switch exhibits the third (high,high) mode and the authors proposed SPK as a contributing mechanism [66], a behavior predicted by our results.

### Interconnected toggle switches

**Fig 6:**
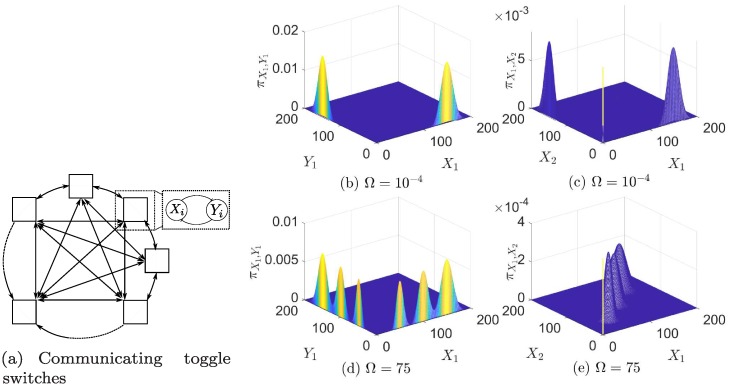
**SPK lead to the emergence of a multi- modal toggle switch** (a) A diagram of population of toggle switches. Arrows between blocks represent reversible diffusion reactions. Each block contains a toggle switch. The remaining subfigures show stationary PMFs for a population of three identical cooperative toggle switches. Due to the symmetries we plot joint PMFs of *X*_1_, *Y*_1_ and *X*_1_, *X*_2_ only. Subplots (b), (c) depict the uncoupled toggle switches. Note that *X*_1_ and *X*_2_ are not synchronized. Subplots (d), (e) depict a high diffusion case. The toggle switches synchronize into a multi-modal toggle switch. More details are given S1 Text §6.4.

Consider *N* copies of the toggle switch defined in the previous section (we consider switches with identical genes for simplicity). They express proteins X_*i*_, Y_*i*_, *i* = 1, ‥, *N*. Let us assume that the switches are interconnected via the diffusion of the proteins among cells, modeled with a diffusion coefficient Ω as: ${\text{X}}_{i}\overset{\Omega }{\underset{\Omega }{⇌}}{\text{X}}_{j},\;{\text{Y}}_{i}\overset{\Omega }{\underset{\Omega }{⇌}}{\text{Y}}_{j},\;i\neq j,\;i,j=1,‥,N$. We view this model as a simplification of a more complex quorum sensing communication mechanism, in which orthogonal AHL molecules are produced by cells and act as activators of TFs in receiving cells, as analyzed for example in [46]. Fig 6a depicts a block diagram of such a network.

For a deterministic model, there exists a parameter range for which all toggle switches synchronize into bistability for sufficiently high diffusion coefficient [46]. This implies each switch in the network behaves as a bistable switch, and it converges with all the other switches to the same steady-states.

Our aim is to analyze the stochastic model at the limit of SPK and compare it to the deterministic model. This network is not in the form of the class of networks in Fig 1. Nevertheless, we show in S1 Text §4 that our results can be generalized to networks with weakly reversible deficiency zero conditional MCs. There are 4^*N*^ conditional MCs, and using Theorem 3, the stationary PMF is a mixture of 4^*N*^ − 1 Poissons.

Consider now the case of a high diffusion coefficient. We show (see S1 Text §3.5) that as Ω → ∞, *X*_1_, ‥, *X*_*N*_ synchronize in the sense that the joint PMF of *X*_1_, ‥, *X*_*N*_ is symmetric with respect to all permutations of the random variables. This implies that the marginal stationary PMFs $p_{X_{i}},i=1,‥,N$ are identical. Hence, for sufficiently large Ω, the probability mass is concentrated around the region for which *X*_1_, ‥, *X*_*N*_ are close to each other. Consequently, for large Ω we can replace the population of toggle switches with a *single toggle switch* with the *synchronized protein processes* *X*(*t*), *Y*(*t*), which are defined, for the sake of convenience, as *X*(*t*) ≔ *X*_1_(*t*), *Y*(*t*) ≔ *Y*_1_(*t*). Next, we describe the stationary PMF of *X*(*t*), *Y*(*t*).

The state of synchronized toggle switches does not depend on individual promoter configurations, and it depends only on the total number of unbound promoter sites in the network. Hence, the number of modes will drop from 4^*N*^ − 1 to (*N* + 1)^2^ − 1. Note that similar to the single toggle switch, there are modes which have both *X*, *Y* with non-zero copy number. On the other hand, there are many additional modes. Recall that in the case of a single toggle switch, we have tuned the cooperativity ratios such that the modes in which both genes are ON are suppressed. Similarly, the undesired modes can be suppressed by tuning the cooperativity ratio which can be achieved by choosing $\rho_{d_{i}}^{X},\rho_{d_{i}}^{Y},d=0,‥,4^{N}-1$ sufficiently large. In particular, letting the multi-merization ratio *β*/*β*_ → ∞, the weights of modes in the interior of the positive orthant $R_{+}^{2}$ approach zero. In conclusion, for sufficiently high Ω and sufficiently high *β*/*β*_ the population behaves as a *multimodal switch*, which means that the whole network can have either the gene X ON, or the gene Y ON. And every gene can take 2*N* modes which are: {(*ik*_0_/(*Nk*_), 0), (0, *ik*_0_/(*Nk*_)): *i* = 1, ‥, *N*}. Comparing to the low diffusion case, the network will have up to 2^*N*^ − 1 modes with sufficiently high multimerization ratio.

In order to illustrate the previous results, consider a population of three toggle switches (*N* = 3) and cooperativity *n* = 2. For large Ω, the deterministic system bifurcates into bistability This means that all toggle switches converge to the same exact equilibria if Ω is greater than a threshold. In contrast, the modes in the stochastic model of the toggle switches converge *asymptotically* to each other. Hence, we need to choose a threshold for Ω that constitutes “sufficient” synchronization. We define this as the protein processes synchronizing within one copy number. In other words, we require the maximum distance between the modes to be less than 1. It can be shown (see S1 Text §3.5) that Ω has to satisfy: $\Omega \geq \frac{1}{N}\left(k-k_{-}\right)$. In this example, the minimal Ω is 75. The stationary PMF is depicted in Fig 6d. The network has 15 modes, nine of which are in the interior and are suppressed due to cooperativity. In contrast, the deterministic model bifurcates into synchronization for Ω > 0.5. The stable synchronized equilibria are (149.98, 0.02), (0.02, 149.98).

The stochastic model with SPK adds four additional modes at (0, 100), (100, 0), (50, 0), (0, 50). To interpret this, note that the protein processes synchronize while the promoter configurations do not. The high states (150, 0), (0, 150) correspond to the case when all the binding sites are empty. In the case when one binding site is empty, the first gene is active while the second and the third are not. Due to diffusion, the first gene “shares” its expressed protein with the other two genes, which implies that each gene will receive a *third* of the total protein copy numbers produced in the network. A similar situation arises when two binding sites are empty.

### The repressilator

**Fig 7:**
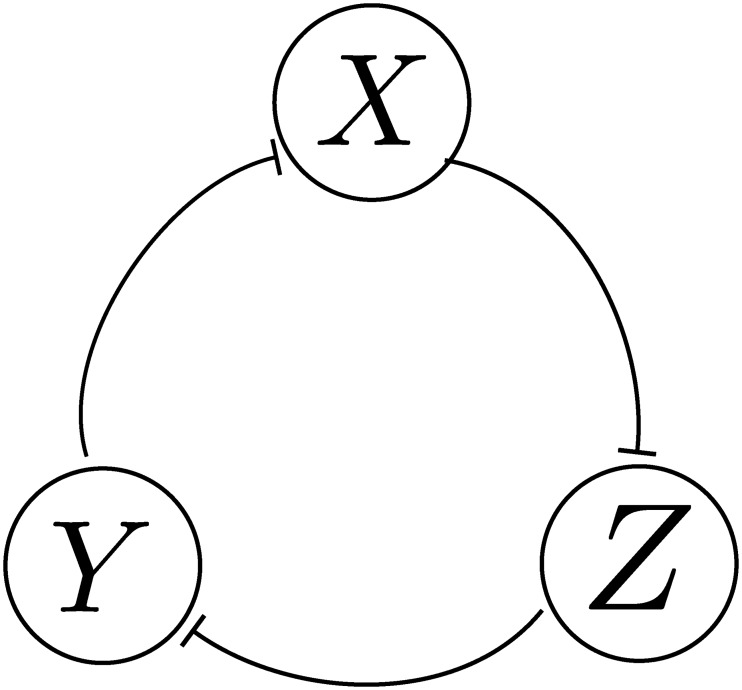
A diagram of the repressilator.

A very different example is provided by a well-studied synthetic oscillator, the repressilator. The repressilator is a synthetic biological circuit that implements a ring oscillator [67], and it has been simulated with slow-promoter kinetics [21]. It is a canonical example of a GRN that exhibits a limit cycle, i.e. sustained oscillation. For simplicity, we study a network consisting of three *identical* genes whose expressed proteins are X, Y, Z. The protein X represses Y, Y represses Z, and Z represses X as shown in Fig 7. Each gene has dissociation ratio *α*/*α*_, production ratio *k*/*k*_ for the unbound state only, multimerization ratio *β*/*β*_, and cooperativity index *n*.

Deterministic analysis of the repressilator [3] reveals that it does not oscillate with non-cooperative binding. black Applying our techniques for the stochastic case with slow promoter kinetics, we are able to find the values of the parameters so that the probability is concentrated in three modes (*K*, 0, 0), (0, *K*, 0), (0, 0, *K*) if *w* ≔ 2(*α*/*α*_)(*k*/*k*_)^*n*^(*β*/(*n*!*β*_)) ≫ 1, where *K* = *k*/*k*_ (see S1 Text §3.4). The obtained tri-modal stationary distribution is consistent with the classical oscillations of the repressilator, and this is independent of the cooperativity index. Note that this condition is analogous to the oscillation condition in the deterministic model [3] (but with cooperativity only) which also requires “large” production ratios.

In order to study whether the network oscillates, we need to define a notion of limit cycle for a stochastic system. Due to randomness, the time-series can not be periodic. Nevertheless, since the stationary distribution is tri-modal, we say that the network oscillates if sample paths (time histories of trajectories) typically jump between the modes in the same order.

Assume *w* ≫ 1. Let *d*_*x*_, *d*_*y*_, *d*_*z*_ be the three dominant modes. We show that if the reduced-order Markov chain is at mode *d*_*x*_ then it is much more likely to transition to *d*_*y*_ rather than to *d*_*z*_. Similar arguments apply if we start from *d*_*y*_, *d*_*z*_. In particular, let $Q\left(t\right)=e^{t\Lambda_{r}}$ be the probability transition matrix. We are interested in comparing the probabilities of transiting from *r*_*x*_ to *r*_*y*_, *r*_*z*_. Hence, we study small *t* ≪ 1. We give expressions for $Q_{d_{x}d_{x}}\left(t\right),Q_{d_{y}d_{x}}\left(t\right),Q_{d_{z}d_{x}}\left(t\right)$ in S1 Text §3.4 which show that if the Markov chain is at *d*_*x*_ then it is most likely to stay there. The transition is much more likely to happen to *d*_*y*_ rather than *d*_*z*_. Hence, we expect to see “long” periods of protein *X* being expressed, and then it jumps to express protein *Y*, and then protein *Z*. Since the finite Markov chain is ergodic, the pattern repeats.

Note that the analysis above predicts that both the cooperative and the noncooperative repressilator are capable of oscillation with slow-promoter kinetics when *w* ≫ 1. The average “period” increases with the production ratio and the transient behavior of the network follows the analysis in S1 Text §5.

**Fig 8:**
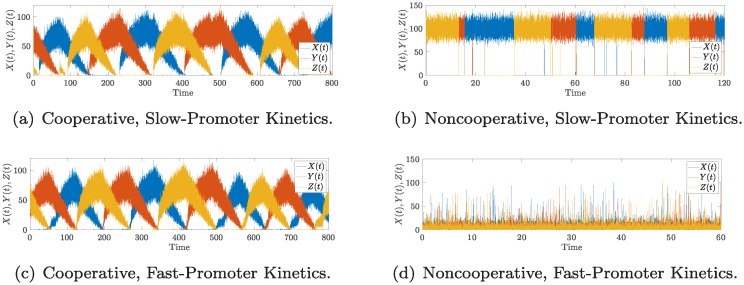
The noncooperative repressilator oscillates. (a) A time-series for the cooperative repressilator with cooperativity index 2, and slow promoter kinetics. (b) A time-series for the cooperative repressilator with cooperativity index 2, and fast promoter kinetics. (c) A time-series for the noncooperative repressilator, and slow promoter kinetics. (d) A time-series for the noncooperative repressilator, and fast promoter kinetics. The plots were generated by stochastic simulation via the Gillespie algorithm. For all the figures, the parameters are: *α* = 5*ε*, *α*_ = 1*ε*, *k* = 2000, *k*_ = 20, *β*_±_ = 1, where *ε* = 0.1 for slow kinetics, and *ε* = 1000 for fast kinetics.

We performed Monte-Carlo simulations via the Gillespie algorithm for both fast and slow kinetics. The results are shown in Fig 8. We observe that the network always oscillates with cooperative binding. With non-cooperative binding, only the network with slow kinetics oscillates, as predicted. The network with fast kinetics does not oscillate. Recall that the deterministic model with non-cooperative binding does not oscillate [3].

### Trans-differentiation network

**Fig 9:**
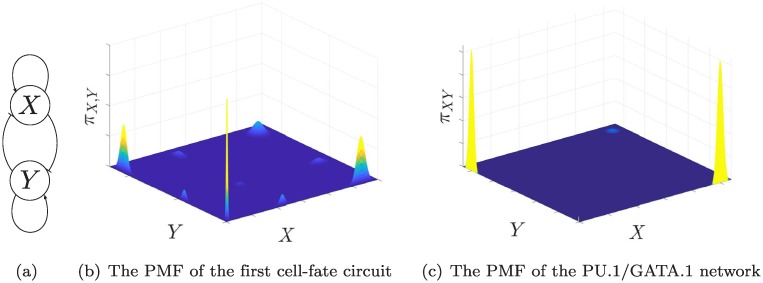
The cell-fate decision network with SPK has more modes than what a deterministic model predicts. (a) A diagram of a generic cell-fate circuit that can describe the networks considered, (b) The PMF of the first cell-fate circuit computed using Theorem 3. (c) The PMF of the PU.1/GATA.1 circuit, where X denotes PU.1 and Y denotes GATA.1. Three modes can be seen. Details are given in S1 Text §6.5.

We consider two networks for TF cross-antagonism in cell fate decision in this section. Both networks consist of two self-activating genes repressing each other as depicted in Fig 9a [5]. The first network [39] has independent cooperative binding of the TFs to the promoters. The genes states are ${\text{D}}_{00}^{X},{\text{D}}_{01}^{X},{\text{D}}_{10}^{X},{\text{D}}_{11}^{X}$ for gene X, and vice versa for gene Y. In order for the genes to be cross-inhibiting and self-activating we let: ${\text{D}}_{01}^{X},{\text{D}}_{10}^{Y}$ have zero production rates. Also, the maximal production rates for genes X, Y occur at gene states ${\text{D}}_{10}^{X},{\text{D}}_{01}^{Y}$, respectively. (See S1 Text §6.5) The network can be analyzed with the proposed framework, as it consists of two genes each with two binding sites. Hence it can theoretically admit up to 16 modes according to (10). The PMF is depicted in Fig 9b for an example parameter set. Note that despite the fact that we have 16 modes, only eight of them contribute to most of the stationary PMF. This is to be contrasted with a deterministic model, which cannot produce more than 4 stable equilibria [52].

The second network that we study is a model of the PU.1/GATA.1 network, which is a lineage determinant in hematopoietic stem cells [68]. Diagrammatically, it can also be presented by Fig 9-a. However, it differs from the first network presented above in several ways. First, PU.1 needs GATA.1 to bind to the promoter of GATA.1 [69], and vice versa [70]. In our modelling framework this means that the promoter configurations ${\text{D}}_{01}^{X},{\text{D}}_{10}^{Y}$ do not exist, where X stands for PU.1 and *Y* stands for GATA.1. Hence, the network has 9 gene states. Second, there is no evidence that PU.1 and GATA.1 form dimers to activate their own promoters cooperatively. In fact, it has been shown that self-activation for GATA-1 occurs primarily through monomeric binding [71]. Further discussion of the model is included in S1 Text §6.5, and is further discussed in [72].

With lack of cooperativity, the deterministic model is only monostable and cannot explain the emergence of bistability for the above network. However, using our framework, up to nine modes can be realized. In order to simplify the landscape, we group the nine into four modes. This is possible since the states ${\text{D}}_{11}^{X},D_{00}^{X}$, $D_{11}^{Y},D_{00}^{Y}$ have very low production rates. This gives a total of *four* modes which are (low,low),(high,low),(low,high),(high,high). Using our model, we choose the parameters to realize bistability and tristability. Fig 9c depicts the stationary PMF for a set of parameters that satisfies the assumptions and give rise to a tristable PMF.

## Methods

**Numerical Simulation Software** Calculations were performed using MATLAB 9. Bertini 1.5 was used for the computation of deterministic solutions of the “quorum sensing” numerical example.

## Discussion

Phenotypical variability in the absence of genetic variation is a phenomenon of great interest in current biological and translational research, as it plays an important role in processes as diverse as embryonic development [73], hematopoietic cell differentiation [74], and cancer heterogeneity [75]. A conceptual, and often proposed, unifying framework to explain non-genetic variability is to think of distinct phenotypes as multiple “metastable states” or “modes” in the complex energetic landscape associated to an underlying GRN. Following this point of view, we studied a general but simplified mathematical model of gene regulation. Our focus has been on stochastic SPK, the time scale relevant when transcription factor binding and unbinding are affected by epigenetic processes such as DNA methylation and chromatin remodeling. In that regime, adiabatic approximations of promoter kinetics are not appropriate. In contrast to the existing literature, which largely confines itself to numerical simulations, in this work we provided a rigorous analytic characterization of multiple modes.

The general formal approach that we developed provides insight into the relative influence of model parameters on system behavior. It also allows making theoretical predictions of how changes in wiring of a GRN, be it through natural mutations or through artificial interventions, impact the possible number, location, and likelihood, of alternative states. We were able to tease out the role of cooperative binding in stochastic models in comparison to deterministic models, which is a question of great interest in both the analysis of natural systems and in synthetic biology engineering. Specifically, we found that, unlike deterministic systems, the number of modes is independent of whether the TF-promoter binding is cooperative or not; on the other hand, cooperative binding gives extra degrees of freedom for assigning weights to the different modes. Emergence of bimodality in noncooperative single gene networks in different contexts has been reported in [76], which studies exogenous TF and fast promoter kinetics, and [77], which studies the effect of temperature fluctuations. The intermediate promoter kinetic domain has been studied in [78]. Switching behavior in a single gene driven by a bursty exogenous input has been studied in [79]. More generally, we characterized the stationary PMFs of CMEs for our GRNs as mixtures of Poisson PMFs, which enabled us to obtain explicit formulas for the locations and probabilities of metastable states as a function of the parameters describing the system.

Although we formulate our study in terms of stationary PMFs, one may equally well view our results as describing the typical dynamic behavior of realizations of the stochastic process. These recapitulate the form of the stationary PMFs: modes are reflected in metastable states along sample paths, states in which the system will stay for prolonged periods until switching to other states corresponding to alternative modes. In the SI, we provide Monte-Carlo simulations showing such metastable behavior along sample paths. We do so for the toggle switch as well as for a version of a well-studied genetic circuit [67] which exhibits oscillatory behavior along sample paths even though the corresponding deterministic model cannot admit oscillations. One application of our mathematical results was to models of single or communicating “toggle switches” in bacteria, where we showed that, for suitable parameters, there are a very large number of metastable attractors.

This work was in fact motivated by our interest in hematopoietic cell differentiation, and in this paper we discussed two possible models of trans-differentiation networks in mammalian cells. In a first model, based on previous publications, we uncovered more modes than had been predicted with different analyses of the same model. This implies that in practice there could be unknown “intermediate” phenotypes that result from the network’s dynamics, which may be acquired by cells during the natural differentiation process or which one might be able to induce through artificial stimulation. The second model included only binding reactions that have been experimentally documented, and as such might be more biologically realistic than the first model. For this second model, a deterministic analysis predicts monostability, which is inconsistent with the fact that the network should control a switch between two stable phenotypes (erythroid and myeloid). This suggests that stochasticity, likely due to low copy numbers and/or SPK, might be responsible for the multiple attractors (phenotypes) that are possible in cell differentiation GRNs. Our mathematical results, being quite generic, should also be useful in the analysis of networks that have been proposed for understanding aspects of cancer biology. For example, non-genetic heterogeneity has been recently recognized as an important factor in cancer development and resistance to therapy, with stochastic multistability in gene expression dynamics acting as a generator of phenotype heterogeneity, setting a balance between mesenchymal, epithelial, and cancer stem-cell-like states [80] [81] [82] [83], and nongenetic variability due to multistability arising from mutually repressing gene networks has been proposed to explain metastatic progression [84].

Application of the results to practical problems entails deciding whether *ε* is small enough. Since singular perturbations rely on a first-order approximation of the stationary PMF (8), the exact determination of the range of *ε* requires determining coefficients of higher-order terms, which can be estimated by computing the asymptotic expansion for a finite state projection of the specific problem at hand. Nevertheless, we provide a simple intuitive rule. The predicted behavior is expected to emerge when the largest rate in the reduced matrix *ε*Λ_*r*_ is slower than the decay rate of proteins. Recall that the elements of Λ_*r*_ depend on the association and dissociation constants and the conditional expectation of protein copy numbers given in (12) as seen in (11). Our numerical examples depicted in Figs 1, 3 and 4, SI1 examine how the approximation fares with multiple levels of time scale separation and agree with the rule. In particular, the latter figure provides Monte-Carlo simulations depicting the third mode of the toggle switch with a two-to-one scale separation per the definition above. Furthermore, since (12) can be tuned by the multimerization ratio, we note that the ratio can make the network more “robust” or vulnerable with respect to the emergence of modes predicted in the SPK regime. In practice, it may be difficult to estimate experimentally the average time that a TF of interest takes to find its binding targets. Hence, we suggest that our results should be considered if there is a very low number of gene copies (i.e., 1-5) and it is suspected that TF-gene binding kinetics are slower than protein kinetics, which may happen particularly in Eukaryotic cells as discussed in the introduction. Our approach can be seen as an addition to the toolbox for analysis of the spectrum of possible behaviors in GRNs, and it can explain apparent multi-modality when the deterministic model can’t. As an example, the recent experimental work on the toggle switch [66] which validated the observation of a third mode and proposed slow promoter kinetics as a mechanism, is consistent with our results.

## Supporting information

S1 Text Supporting information file with mathematical proofs, detailed analysis of examples, generalization of the results and additional simulations. (PDF)
