# Supplementary material for: Multi-modality in gene regulatory networks with slow promoter kinetics
Source: PLoS Comput Biol. 2019 Feb 19;15(2):e1006784. doi: 10.1371/journal.pcbi.1006784 (PMC6396950; doi:10.1371/journal.pcbi.1006784)
Supplement: S1 Text — (PDF) [file pcbi.1006784.s001.pdf]

Supplementary Information to “Multi-modality in gene regulatory networks with slow promoter kinetics”

M. Ali Al-Radhawi[2], Domitilla Del Vecchio[1], and Eduardo D. Sontag[2, 3]

[1]Department of Mechanical Engineering, Massachusetts Institute of Technology, Cambridge, MA 02139-4307, USA. Emails: {malirdwi,ddv}@mit.edu.
[2]Departments of Bioengineering and of Electrical and Computer Engineering, Northeastern University, Boston, MA 02115. Email: sontag@sontaglab.org
[3]Institute of Systems Pharmacology, Harvard Medical School, Boston, MA 02115.

1 Review of the Master Equation and Markov Chains

1.1 The Chemical Master Equation

A generic reaction $\mathrm{R}_j \in \mathscr{R}$ takes the form:

$$\mathrm{R}_j : \sum_{i=1}^{|\mathscr{S}|} \alpha_{ij} \mathrm{Z}_i \to \sum_{i=1}^{|\mathscr{S}|} \beta_{ij} \mathrm{Z}_i, \tag{1}$$

where $Z_i \in \mathscr{S}$, α_{ij}, and β_{ij} are positive integers. The reactions that we consider are limited to at most two reactants. The reverse reaction of R_j is the reaction in which the products and reactants are interchanged. If the network contains both the reaction and its reverse then we use the short-hand notation to denote both of them as

$$\mathrm{R}_j : \sum_{i=1}^{|\mathscr{S}|} \alpha_{ij} \mathrm{Z}_i \rightleftharpoons \sum_{i=1}^{|\mathscr{S}|} \beta_{ij} \mathrm{Z}_i. \tag{2}$$

The stoichiometry of a CRN can be summarized by a *stoichiometry matrix* Γ which is defined element-wise as follows:

$$[\Gamma]_{ij} = \beta_{ij} - \alpha_{ij}.$$

The columns of the stoichiometry matrix $\gamma_1, .., \gamma_{|\mathscr{R}|}$ are known as the stoichiometry vectors. We say that a nonzero nonnegative vector d gives a *conservation law* for the stoichiometry if $d^T \Gamma = 0$.

Kinetics:
The kinetics of the network quantify the speed of transformation from reactants into products whenever a reaction occurs. In order to keep track of molecule counts, each species $\mathrm{Z}_i \in \mathscr{S}$ is associated with a copy number $z_i \in \mathbb{Z}_{\geq 0}$.

To each reaction R_j one associates a propensity function R_j. Assuming a homogeneous well-stirred isothermal medium with a fixed volume, the most common model of propensities, which we use, is the *Mass-Action Kinetics* which is derived from the principle that the likelihood of two

reactant molecules colliding and reacting is proportional to their copy numbers. If R_j has a single reactant species Z_i with stoichiometry coefficient α_{ij}, then [1]:

$$R_j(z_i) = k_j \binom{z_i}{\alpha_{ij}} = k_j \frac{z_i(z_i-1)...(z_i-\alpha_{ij})}{\alpha_{ij}!},$$

where k_j is a kinetic rate constant. Note if $\alpha_{ij}=1$, then $R_j(z_i)=k_j z_i$.

Dynamics:
The dynamics of the network refers to the manner in which the *state* evolves in time, where the state $Z(t) \in \mathbf{Z} \subset \mathbb{Z}_{\geq 0}^{|\mathscr{S}|}$ is the vector of copy numbers of the species of the network at time t. Since the collision of molecules is random in nature, the time-evolution of states is described mathematically by a stochastic process. The standard stochastic model for a CRN is that of a continuous Markov chain. Let $\mathbf{Z}$ denote the state space. Consider a time t and let the state be $Z(t)=z\in\mathbf{Z}$. Then, the probability that the jth reaction fires in an interval $[t, t+\delta]$ is $R_j(z)\delta + o(\delta)$. If R_j fires, then the states changes from z to $z+\gamma_j$, where γ_j is the corresponding stoichiometric vector.

As Z is a stochastic process we are interested in characterizing its qualitative behavior given by the joint probability distribution $p_z(t)=\Pr[Z(t)=z|Z(0)=z_0]$ for any given initial condition z_0. The time-evolution of the probability distribution can be shown [1] to be given by a system of linear ordinary differential equations known as the *forward Kolmogorov equation* or the *Chemical Master Equation*, given by:

$$\dot{p}_z(t) = \sum_{j=1}^{|\mathscr{R}|} R_j(z-\gamma_j)p_{z-\gamma_j}(t) - R_j(z)p_z(t), z\in\mathbf{Z}, \tag{3}$$

where $\gamma_1, .., \gamma_{|\mathscr{R}|}$ are the columns of the stoichiometry matrix.

Since our species are either gene species or protein species, we split the stochastic process $Z(t)$ into two subprocesses: *the gene process* $D(t)$ and *the protein process* $X(t)$, as explained below.

Consider the ith gene. For each configuration species $\mathrm{D}_j^i \in B_i$, let $D_j^i(t) \in \{0,1\}$ denote its occupancy, i.e. if $D_j^i(t)=1$, then at time t the ith gene is in a configuration $j\in B_i$. It can be seen from gene reactions that the network always has a conservation law supported on $\{\mathrm{D}_j^i, j\in B_i\}$, so that:

$$\sum_{j\in B_i} D_j^i(t) = 1,$$

which reflects the physical constraint that the promoter can be in only one configuration at any given time.

This conservation law enables us to introduce an equivalent reduced representation. For each gene we define one process D_i such that $D_i(t)\in B_i$. $D_i(t)=j$ if and only if $D_j^i(t)=1$. Collecting these into a vector, define the gene process $D(t) := [D_1(t), ..., D_N(t)]^T$ where $D(t)\in\prod_{i=1}^N B_i$. The ith gene can be represented by $|B_i|$ states, so $L := \prod_{i=1}^N |B_i|$ is the total number of promoter configurations in the GRN. With abuse of notation, we write also $D(t)\in\{0, .., L-1\}$ in the sense of the bijection between $\{0, .., L-1\}$ and $\prod_{i=1}^N B_i$ defined by interpreting $D_1...D_N$ as a binary representation of an integer. Hence, $d\in\{0, .., L-1\}$ corresponds to $(d_1, ..., d_N)\in B_1\times .. \times B_N$ and we write $d=(d_1, .., d_N)$.

Since each gene expresses a corresponding protein, we define $X_{i1}(t)\in\mathbb{Z}_{\geq 0}, i=1, .., N$ protein processes. If the multimerized version of the ith protein participates in the network as an activator or repressor then we define $X_{ic}(t)$ as the corresponding multimerized protein process, and we denote

$X_i(t) := [X_{i1}(t), X_{ic}(t)]^T$. If there is no multimerization reaction then we define $X_i(t) := X_{i1}(t)$. Since not all proteins are necessarily multimerized, the total number of protein processes is $N \leq M \leq 2N$. Hence, the *protein process* is $X(t) = [X_1^T(t), .., X_N^T(t)]^T \in \mathbb{Z}_{\geq 0}^M$ and the state space can be written as $\mathbf{Z} = \mathbb{Z}_{\geq 0}^M \times \prod_{i=1}^N B_i$.

Consider the joint probability distribution:

$$p_{d,x}(t) = \Pr[X(t) = x, D(t) = d], \tag{4}$$

which represents the probability at time t that the protein process X takes the value $x \in \mathbb{Z}_+^M$ and the gene process D takes the value $d \in \{0, .., L-1\}$. Recall that x is a vector of copy numbers for the protein processes while d encodes the configuration of each promoter in the network. Then, we can define for each fixed d:

$$p_d(t) := [p_{dx_0}(t), p_{dx_1}(t), ...]^T, \tag{5}$$

representing the vector enumerating the probabilities (4) for all values of x and for a fixed d, where $x_0, x_1, ..$ is an indexing of $\mathbb{Z}_{\geq 0}^M$. Note that $p_d(t)$ can be thought of as an infinite vector with respect to the aforementioned indexing. Finally, let

$$p(t) := [p_0(t)^T, ..., p_{L-1}^T(t)]^T \tag{6}$$

representing a concatenation of the vectors (5) for $d = 0, .., L-1$. Note that $p(t)$ is a finite concatenation of infinite vectors.

The joint stationary distribution $\bar{\pi}$ is defined as the following limit, which we assume to exist and be independent of the initial distribution:

$$\bar{\pi} = \lim_{t \to \infty} p(t). \tag{7}$$

The stationary distribution $\bar{\pi}$ is a function of ε also.

Consider a given GRN. The master equation (3) is defined over a countable state space $\mathbf{Z}$ which can be enumerated with an arbitrarily chosen order. Hence, the master equation can be interpreted as an infinite system of differential equations. Its infinite infinitesimal generator matrix Λ can be written succinctly entry-wise as:

$$\lambda_{z\tilde{z}} := \begin{cases} R_j(z) & \text{if } \exists j \text{ such that } \tilde{z} = z - \gamma_j \\ -\sum_{\tilde{z} \neq z} \lambda_{z\tilde{z}} = -\sum_{j=1}^{|\mathscr{R}|} R_j(z) & \text{if } \tilde{z} = z \\ 0 & \text{otherwise} \end{cases}, \tag{8}$$

where $\lambda_{z\tilde{z}}$ refers to the rate of transition from z to $\tilde{z}$. The matrix Λ is stochastic, which means that it is Metzler and $1^T \Lambda = 0$. A Metzler matrix is a matrix whose off-diagonal elements are non-negative.

1.2 Irreducibility

An important property in the context of Markov chain analysis is that of *irreducibility* [2], and its significance stems from the fact that it is a necessary condition for the existence of a unique positive stationary distribution. Consider the Markov chain $Z(t)$ defined on $\mathbf{Z}$ with an associated infinitesimal generator Λ as given in (8). Let $z, w \in \mathbf{Z}$. Then, it is said that z leads to w if there exist states $z_0, ..., z_n \in \mathbf{Z}$ such that $\lambda_{zz_0}\lambda_{z_0z_1}...\lambda_{z_nw} > 0$. A set $U \subset \mathbf{Z}$ is said to be a *communicating class* if for every $z_1, z_2 \in U$, z_1 leads to z_2 and z_2 leads to z_1. The state space $\mathbf{Z}$ can always be partitioned into a disjoint union of communicating classes [2]. The Markov chain is said to be

irreducible if the state space is a *communicating class*. A communicating class U is said to be *closed* if $z \in U$, and z leads to w implies $w \in U$. A Markov chain is said to be *weakly irreducible* if it has a unique closed communicating class U, and for all $z \in \mathbf{Z}$, z leads to some element U.

We state the following result, under assumption A4:

Proposition SI-1. *Consider a gene regulatory network that consists of N gene expression blocks. Then the associated Markov chain is weakly irreducible.*

Proof. Consider the state $0 \in \mathbf{Z}$. We first show that for all $z \in \mathbf{Z}$, z leads to 0. Let $z = (x_1, d_1, .., x_N, d_N)$. We list the set of reactions, i.e transitions, that will lead to 0. Consider $d_i \neq 0$, if $d_i = 1$ then we apply either the reaction

$$\mathrm{D}_1^i \xrightarrow{\alpha_{-i}} \mathrm{D}_0^i. \tag{9}$$

or

$$\mathrm{D}_1^i \xrightarrow{\alpha_{-i}} \mathrm{TF} + \mathrm{D}_0^i. \tag{10}$$

If $d_i = 10$, then we apply

$$\mathrm{D}_{10}^i \xrightarrow{\alpha_{-i1}} \mathrm{TF}_1 + \mathrm{D}_{00}^i \tag{11}$$

and if $d_i = 01$ we apply

$$\mathrm{D}_{01}^i \xrightarrow{\alpha_{-i3}} \mathrm{TF}_2 + \mathrm{D}_{00}^i. \tag{12}$$

If $d_i = 11$, then we apply two consecutive reactions

$$\mathrm{D}_{11}^i \xrightarrow{\alpha_{-i2}} \mathrm{TF}_1 + \mathrm{D}_{01}^i \tag{13}$$

$$\mathrm{D}_{01}^i \xrightarrow{\alpha_{-i3}} \mathrm{TF}_2 + \mathrm{D}_{00}^i. \tag{14}$$

Hence, z leads to a state of the form $(x_1, 0, x_2, 0, ..., x_N, 0)$. Similarly, we can apply the decay reaction

$$\mathrm{X}_i \xrightarrow{k_{-i}} \emptyset. \tag{15}$$

and the reverse dimerization until we reach the origin.

Now we show that there exists a closed communicating class. If 0 does not lead to any state then $\{0\}$ is a closed communicating class. Otherwise, let U be the smallest communicating class containing 0. Note that U is closed, since if there exists $z \in U$ that leads to w, then w leads to $0 \in U$.

In order to show that U is unique, assume that there exists another closed communicating class U'. But this contradicts with the fact that all $z \in U'$ lead to $0 \in U$. We have shown that for all $z \in \mathbf{Z}$, z leads to 0. Hence z leads to U. □

Remark SI-1. *For finite Markov chains, weak irreducibility with appropriate stochastic stability assumptions are sufficient for the existence of a* nonnegative *unique stationary distribution [3], while irreducibility is usually needed for the existence a* positive *stationary distribution. Note that not all GRNs are irreducible. However, our subsequent results require weak irreducibility only, and investigation of irreducibility is out of the scope of this paper. Nevertheless, necessary and sufficient graphical conditions for irreducibility can be developed and are subject to future work.*

2 Proofs of the Main Results

In this section we include mathematical proofs of the main results in the main text.

2.1 Decomposition of the Master Equation

We include a proof for Proposition 1.

By the time-scale separation assumption, the gene reactions are slow and the protein reactions are fast. Then (8) can be written as:

$$\lambda_{z\tilde{z}} := \begin{cases} R_j^{(f)}(z) + \varepsilon R_j^{(s)}(z) & \text{if } \exists j \text{ such that } \tilde{z} = z - \gamma_j \\ -\sum_{\tilde{z}\neq z} \lambda_{z\tilde{z}} = -\sum_{j=1}^{|\mathscr{R}|} R_j^{(f)}(z) + \varepsilon R_j^{(s)}(z) & \text{if } \tilde{z} = z \\ 0 & \text{otherwise} \end{cases}, \tag{16}$$

where $(f), (s)$ denote fast and slow, respectively.

Hence, the summation in (3) can decomposed into two terms. This implies that the system matrix can be written as a sum of a fast matrix $\tilde{\Lambda}$ and a slow matrix $\varepsilon\hat{\Lambda}$ as in Eq. (2).

We now show that Eq. (3) holds, which amounts to showing that $\tilde{\Lambda}$ is block diagonal. Assume $\exists j$ such that $\tilde{z} = z - \gamma_j$. Let $z = (x, d), \tilde{z} = (\tilde{x}, \tilde{d})$ with $\tilde{d} \neq d$. As can be seen in Figure 2, protein reactions do not change the promoter configuration state d. Hence, the transition rate $\lambda_{z\tilde{z}}$ has terms corresponding to gene reactions rate only, i.e., $\lambda_{z\tilde{z}} = \varepsilon R_j^{(s)}(z)$. Hence, $\tilde{\Lambda}$ is block diagonal. ■

2.2 Analytic Expression of the Conditional Probability Distributions

We include a proof of Proposition 2.

As mentioned before, the stationary distribution is the product of the marginal stationary distributions, since the underlying conditional stochastic processes are independent. If X_i does not form a multimer then it is known that the stationary distribution of the reaction network (54) is Poisson with mean k_{id_i}/k_{-i} as in (51),

Assume, instead, that X_i forms a multimer. In order to simplify notations, we drop the index i and write $\emptyset \underset{k_-}{\overset{k}{\rightleftharpoons}} \mathrm{X}$, $n\mathrm{X} \underset{\beta_-}{\overset{\beta}{\rightleftharpoons}} \mathrm{X}_n$. Let x_1, x_2 denote the molecular counts of X, X_n. Then, the master equation is

$$\dot{p}_{x_1,x_2} = (kp_{x_1-1,x_2} - k_-x_1p_{x_1,x_2}) + (k_-(x_1+1)p_{x_1+1,x_2} - kp_{x_1,x_2}) \tag{17}$$

$$+ \left(\beta_{-2}(x_2+1)p_{x_1-2,x_2+1} - \tfrac{1}{n!}\beta \prod_{k=0}^{n-1}(x_1-k)p_{x_1,x_2}\right) \tag{18}$$

$$+ \left(\tfrac{1}{n!}\beta \prod_{k=1}^{n}(x_1+k)p_{x_1+n,x_2-1} - \beta_-x_2p_{x_1,x_2}\right).. \tag{19}$$

We solve the recurrence equation assuming detailed balance, and then verify that the obtained solution, which is given in (51), solves (17) ■

2.3 The Stationary Distribution as a Mixture of Poisson Distributions

We include here the proof of Theorem 3.

Recall the slow-fast decomposition of the master equation in Eq. (2). Recall the joint stationary distribution (7). In order to emphasize the dependence on ε we denote $\bar{\pi}^\varepsilon := \bar{\pi}(\varepsilon)$. Hence, $\bar{\pi}^\varepsilon$ is the unique stationary distribution that satisfies $\Lambda_\varepsilon\bar{\pi}^\varepsilon = 0$, $\pi^\varepsilon > 0$, and $\sum_z \pi_z^\varepsilon = 1$, where the subscript denotes the value of the stationary distribution at z.

Our objective is to characterize the stationary distribution as $\varepsilon \to 0$. Writing $\bar{\pi}_\varepsilon$ as an asymptotic expansion to first order in terms of ε, we have

$$\bar{\pi}^\varepsilon = \bar{\pi}^{(0)} + \bar{\pi}^{(1)}\varepsilon + o(\varepsilon). \tag{20}$$

Our aim is to find $\bar{\pi}^{(0)}$. Substituting $\bar{\pi}^{\varepsilon}$ in Eq. (2), and equating the coefficients of the powers of ε to zero we obtain the following two equations:

$$\tilde{\Lambda}\bar{\pi}^{(0)} = 0 \tag{21}$$

$$\tilde{\Lambda}\bar{\pi}^{(1)} + \hat{\Lambda}\bar{\pi}^{(0)} = 0 \tag{22}$$

where $\tilde{\Lambda}$ is given in Eq. (3). (21) implies that $\bar{\pi}^{(0)} \in \ker \tilde{\Lambda}$, where ker denotes the kernel of $\tilde{\Lambda}$. We next show how to compute $\ker \tilde{\Lambda}$.

Recall the conditional Markov chains with the associated infinitesimal generators as in Eq. (4). By the assumptions, for each $d \in \{0, .., L-1\}$ there exists a unique $\pi_{X|d}$ such that: $\Lambda_d \pi_{X|d} = 0$, $\pi_{X|d} > 0$, and $\sum_x \pi_{X|d}(x) = 1$. Recall that $\pi_{X|d}$ is the stationary distribution of the Markov chain conditioned on $D(t) = d$.

Defining the extended conditional distributions for $d = 0, .., L-1$ as:

$$\bar{\pi}_{X|d} := [\overbrace{\mathbf{0}^T \; ... \; \mathbf{0}^T}^{d-1} \, \pi_{X|d}^T \, \overbrace{\mathbf{0}^T \; ... \; \mathbf{0}^T}^{L-d}]^T. \tag{23}$$

The stationary distribution above can be interpreted as a function as follows: $\bar{\pi}_{X|d}(x, d) = \pi_{X|d}(x)$, and $\bar{\pi}_{X|d}(x, d') = 0$ when $d' \neq d$.

Then $\ker \tilde{\Lambda} = \text{span}\{\bar{\pi}_{X|0}, .., \bar{\pi}_{X|L-1}\}$. Hence, we can write:

$$\bar{\pi}^{(0)} = \sum_{i=0}^{L-1} \lambda_i \bar{\pi}_{X|i},$$

for some $\lambda_0, ..., \lambda_{L-1} \geq 0$. We normalize them to satisfy $\sum_{d=0}^{L-1} \lambda_d = 1$.

In order to satisfy (22), we utilize the fact that each Λ_d is an infinitesimal generator which satisfies $\mathbf{1}^T \Lambda_d = 0$. Hence, we pre-multiply (22) by the vectors: $[\mathbf{1}^T \; \mathbf{0}^T \; ...\mathbf{0}^T]^T$, $[\mathbf{0}^T \; \mathbf{1}^T \; ...\mathbf{0}^T]^T$, $[\mathbf{0}^T \; \mathbf{0}^T \; ... \; \mathbf{1}^T]^T$ in order to get the following L-dimensional linear system:

$$\Lambda_r \lambda := \begin{bmatrix} \mathbf{1}^T & \mathbf{0}^T & ... & \mathbf{0}^T \\ \mathbf{0}^T & \mathbf{1}^T & ... & \mathbf{0}^T \\ & & \ddots & \\ \mathbf{0}^T & \mathbf{0}^T & ... & \mathbf{1}^T \end{bmatrix} \hat{\Lambda} \, [\bar{\pi}_{X|0} \; \bar{\pi}_{X|1} \; ... \; \bar{\pi}_{X|L-1}] \begin{bmatrix} \lambda_0 \\ \vdots \\ \lambda_{L-1} \end{bmatrix} = 0. \tag{24}$$

Furthermore, we need the following normalization equation to find $\lambda_0, ..., \lambda_{L-1}$ uniquely:

$$\lambda_0 + ... + \lambda_{L-1} = 1. \tag{25}$$

This is equivalent to stating that $\lambda = [\lambda_0, ..., \lambda_{L-1}]$ is the principal eigenvector of Λ_r. ■

2.4 Computation of the Reduced-Order Markov Chain's Generator

Recall that the generator of the reduced-order Markov chain can be written as follows:

$$\Lambda_r = \begin{bmatrix} \mathbf{1}^T \hat{\Lambda}_{00} \pi_0 & \dots & \mathbf{1}^T \hat{\Lambda}_{0L-1} \pi_{L-1} \\ \vdots & \ddots & \\ \mathbf{1}^T \hat{\Lambda}_{L-10} \pi_0 & \dots & \mathbf{1}^T \hat{\Lambda}_{(L-1)(L-1)} \pi_{L-1} \end{bmatrix}. \tag{26}$$

The (d', d) entry represents the probability of transition from the configuration d' to configuration d, and it can be interpreted as a weighted conditional expectation of π_d.

Consider the reduced chain, and fix a configuration d. Then, the maximum number of possible transitions out of d is given by the number of reactions which is $\frac{1}{2}\sum_{i=1}^{N}|B_i|$. Hence, Λ_r is a sparse matrix for large N. Computation of the infinite matrices and matrix product in (26) can be cumbersome for networks with multiple genes. Hence, we provide an algorithm for computing the nonzero entries in Λ_r. This can be achieved by considering all the possible transitions from a configuration $d = (d_1, .., d_N)$. Specifically, we consider a transition from d to d' by a gene reaction modifying a single promoter configuration. For instance consider D_{d_i}. Then for a constitutive or single TF-gene binding/unbinding, there can be only one transition starting from D_{d_i}. This transition is either the forward or reverse reaction with an autonomous or single TF expression. For the case of two TFs, there can be two reactions among the possible four gene reactions.

The algorithm can be described as follows:

Proposition SI-2. *The matrix Λ_r in* (24) *can be computed via the algorithm below.*

- *For each $d \in \{0, .., L-1\}$ write $d = (d_1, .., d_N) \in \prod_{i=1}^{N} B_i$. Using the previously discussed identification:*
  - *Let $\mathscr{R}_d = \{\mathrm{R}_1, .., \mathrm{R}_{|\mathscr{R}_\mathrm{d}|}\}$ the set of all gene reactions. Then, for each $j \in \{1, .., |\mathscr{R}_d|\}$:*
    1. *Let $D^i_{d_i}$, and $D^i_{d_{i'}}$ be the reactant and product configuration species of the* $\mathrm{R_j}$*. Hence, the reaction will cause a transition from d to $d' = (d_1, .., d_{i'}, .., d_N)$. Let α be the kinetic constant of* $\mathrm{R_j}$*. If* $\mathrm{R_j}$ *is a binding reaction, then let $\mathrm{X}_{\bar{i}}$, $\mathrm{X}_{\bar{i}c}$ denote the TF or the multi-merized TF, where $\bar{i}$ denotes the index of the gene that expresses the TF.*
    2. *Then, the (d', d) entry of Λ_r can be written as:*

$$[\Lambda_r]_{d'd} = \begin{cases} \alpha, & \text{if the reaction is monomolecular} \\ \frac{\alpha}{n_{\bar{i}}!}\frac{\beta_{\bar{i}}}{\beta_{-\bar{i}}}\left(\frac{k_{\bar{i}d_{\bar{i}}}}{k_{-\bar{i}}}\right)^{n_{\bar{i}}}, & \text{if the reaction is bimolecular} \end{cases} \tag{27}$$

  - *Set*

$$[\Lambda_r]_{dd} = -\sum_{i' \neq i} \mathbf{1}^T \hat{\Lambda}_{d_{i'}d_i}\pi_d. \tag{28}$$

- *Set the rest of the entries of Λ_r to zero.*

Proof. Recall that in Eq. (2), the matrix $\hat{\Lambda}$ represents the slow matrix, which corresponds to the gene binding reactions. Hence, $\hat{\Lambda}_{dd'}$ represents the matrix corresponding to the transition between states of the form (x, d) and (x, d'). Assume that $D(t) = d = (d_1, ..., d_N)$. Consider the ith block. Note that it has one or two reactions that can fire. Specifically, there are $\frac{1}{2}|B_i|$ gene reactions that can fire. Assume that such a reaction is in one of the forms:

$$\mathrm{D}^i_{d_i} \xrightarrow{\alpha} \mathrm{D}_{d_{i'}}, \mathrm{D}^i_{d_i} \xrightarrow{\alpha}\xrightarrow{\alpha} \mathrm{D}_{d_{i'}} + \mathrm{X}_{\bar{i}},$$

where $\mathrm{X}_{\bar{i}}$ is a TF for that block. Then,

$$[\Lambda_r]_{d'd} = \mathbf{1}^T\Lambda_{d'd}\pi_d = \alpha\mathbf{1}\pi_d = \alpha.$$

Now consider a reaction of the form:

$$\mathrm{X}_{\bar{i}} + \mathrm{D}^i_{d_i} \xrightarrow{\alpha} \mathrm{D}^i_{d_{i'}}.$$

Then,

$$[\Lambda_r]_{d'd} = \mathbf{1}^T \Lambda_{d'd} \pi_d = \alpha \sum_{x_{\bar{i}}=0}^{\infty} x_{\bar{i}} \pi_{d\bar{i}} \sum_{x_{i\neq\bar{i}}} \pi_{di} = \alpha \mathbb{E}[X_{\bar{i}}(t)|D(t)=d] = \alpha \frac{k_{\bar{i}d_{\bar{i}}}^{n_{\bar{i}}} \beta_{\bar{i}}}{n_{\bar{i}}! k_{-\bar{i}d_{\bar{i}}}^{n_{\bar{i}}} \beta_{-\bar{i}}}.$$

The last equality follows from evaluating the mean value of the Poisson distribution in (52).

Finally, (28) holds since $\mathbf{1}^T \Lambda_r = 0$, which follows from $\mathbf{1}^T \hat{\Lambda} = 0$. ■

3 Detailed Discussion of Examples

3.1 The Gene Bursting Model

We start with the simplest form of network, which is the autonomous TF-gene binding/unbinding model. It has been verified as a model for transcriptional bursting [4]. This model has been studied analytically using time-scale separation [5],[6], Poisson-representations [7], and the exact steady solution is known [8], [9].

Consider:

$$\begin{aligned} &\mathrm{D}_0 \underset{\varepsilon\alpha_-}{\overset{\varepsilon\alpha}{\rightleftharpoons}} \mathrm{D}_1 \\ &\mathrm{D}_1 \xrightarrow{k} \mathrm{X} + \mathrm{D}_1, \\ &\mathrm{X} \xrightarrow{k_-} 0. \end{aligned} \tag{29}$$

Referring to Figure 2, we identify a single gene block with two states. Using (51), the conditional stationary distributions are:

$$\begin{aligned} \pi_0(x) &= \delta(x) \\ \pi_1(x) &= \mathbf{P}(x; k/k_-). \end{aligned}$$

In order to compute the stationary distribution π, we need to find the generator for the reduced chain. Since both reactions are monomolecular, we write the following using (27):

$$\Lambda_r = \begin{bmatrix} -\alpha & \alpha_- \\ \alpha & -\alpha_- \end{bmatrix}.$$

Hence, the reduced Markov chain is a binary Bernoulli process with a rate of $\alpha/(\alpha+\alpha_-)$. Then the stationary distribution of X can be written using (57) as:

$$\pi(x) = \frac{\alpha_-}{\alpha+\alpha_-} \mathbf{P}(x; 0) + \frac{\alpha}{\alpha+\alpha_-} \mathbf{P}(x; k/k_-), \tag{30}$$

which is a bimodal distribution with peaks at 0 and k/k_-. The fast promoter kinetics model is obtained, instead, by reversing the time-scale separation such that the protein reactions become slow and gene reactions become fast. In that case, the resulting stationary distribution can be shown to be a Poisson with mean $\frac{\alpha}{\alpha+\alpha_-}\frac{k}{k_-}$ which is the same as the deterministic equilibrium if we used the conservation law $D_1(t) + \mathrm{D}_0(t) = 1$ for the above model. Finally, note that the mean of the slow promoter kinetics model is the same as in the fast kinetics model but the two stationary distributions differ drastically.

3.2 A Self-Regulating Gene

Consider a non-cooperative self-regulating gene:

$$
\begin{aligned}
\mathrm{X} + \mathrm{D}_0 &\underset{\varepsilon\alpha_-}{\overset{\varepsilon\alpha}{\rightleftharpoons}} \mathrm{D}_1 \\
\mathrm{D}_0 &\overset{k_0}{\to} \mathrm{D}_0 + \mathrm{X}, \\
\mathrm{D}_1 &\overset{k_1}{\to} \mathrm{D}_1 + \mathrm{X}, \\
\mathrm{X} &\overset{k_-}{\to} 0.
\end{aligned} \tag{31}
$$

Referring to Figure 2, this is a single-gene block with two states. At the limit of slow promoter kinetics, Remark 4 implies that the gene binding/unbinding reaction can be written as as follows:

$$
\mathrm{D}_0 \underset{\alpha_-}{\overset{\alpha k_0/k_-}{\rightleftharpoons}} \mathrm{D}_1.
$$

Using (27), the reduced generator can be written as:

$$
\Lambda_r = \begin{bmatrix} -\alpha k_0/k_- & \alpha_- \\ \alpha k_0/k_- & -\alpha_- \end{bmatrix}.
$$

Hence it defines a binary Bernoulli process with the rate $\alpha k_0/(\alpha k_- + \alpha k_0)$. Using (57) the stationary distribution is a mixture of two Poisson distributions and can be written as:

$$
\pi_1(x) = \frac{\alpha\rho_1}{\alpha_- + \alpha\rho_1}\mathbf{P}(x; k_1/k_-) + \frac{\alpha_-}{\alpha_- + \alpha\rho_1}\mathbf{P}(x; k_0/k_-), \tag{32}
$$

where

$$
\rho_1 = \mathbb{E}[X_2|D=0] = k_0/k_-.
$$

Next, consider the same reaction network, but now with cooperativity:

$$
\begin{aligned}
\mathrm{X}_2 + \mathrm{D}_0 &\underset{\varepsilon\alpha_-}{\overset{\varepsilon\alpha}{\rightleftharpoons}} \mathrm{D}_1 \\
\mathrm{D}_0 &\overset{k_0}{\to} D_0 + \mathrm{X}, \\
\mathrm{D}_1 &\overset{k_1}{\to} \mathrm{D}_1 + \mathrm{X}, \\
\mathrm{X} &\overset{k_-}{\to} 0 \\
2\mathrm{X} &\underset{\beta_-}{\overset{\beta}{\rightleftharpoons}} \mathrm{X}_2.
\end{aligned} \tag{33}
$$

In this case, the gene process is still a Bernoulli process, but with a different rate. The stationary distribution for X can be written as:

$$
\pi_2(x) = \frac{\alpha\rho_2}{\alpha_- + \alpha\rho_2}\mathbf{P}(x; k_1/k_-) + \frac{\alpha_-}{\alpha_- + \alpha\rho_2}\mathbf{P}(x; k_0/k_-), \tag{34}
$$

where

$$
\rho_2 = \mathbb{E}[X_2|D=0] = \frac{k_0^2\beta}{2k_-^2\beta_-}.
$$

Both distributions (32), (34) have modes at $\frac{k_1}{k_-}$ and $\frac{k_0}{k_-}$. The height of the first mode is proportional to ρ_1 for (32), and is proportional to ρ_2 for (34). The network is activating if $k_1 > k_0$, and repressing otherwise.

Comparing (32) and (34), note that, in the non-cooperative case, if we want to increase the weight of the mode corresponding to the bound state keeping the association ratio, then the mode location needs to be changed. On the other hand, the factor ρ_2 in the dimerization rates in (34) can be used in order to tune the weights freely while keeping the modes and the binding to unbinding kinetic constants ratio unchanged. For instance, we can make the distribution effectively unimodal with a sufficiently high dimerization ratio.

A non-cooperative self-regulating gene with slow promoter kinetics has been studied in the literature by deriving closed-form expressions [10], and using time-scale separation [5] . However, the gene binding/unbinding reaction in both of these papers was approximated by an auto-catalytic reaction:

$$\mathrm{X} + \mathrm{D}_0 \underset{\varepsilon\alpha_-}{\overset{\varepsilon\alpha}{\rightleftharpoons}} \mathrm{D}_1 + \mathrm{X}.$$

This has the advantage of decoupling the slow and the fast processes. However, it is a simplification of the physical process. We did not use such simplifications.

Special cases:
The model above considers a network with possibly non-zero production rates for both the unbound and bound promoter configurations. We may also consider the special cases of pure self-activation or self-repression which are explained below:

1. *Pure Self-Activation*, i.e. $k_0 = 0$ in (31) and (33). Then the peak corresponding to the bound configuration disappears and we get only one peak at zero protein copy number as it forms an absorbing state. This is a manifestation of the Keizer's paradox [11]. One way to circumvent this is to allow for a small transcriptional "leak". This amounts to taking $k_0 \ll k_1$, and it allows us to recover the second mode.

2. *Pure Self-Repression*, i.e. $k_1 = 0$ in (31) and (33). Then we get two modes: one at 0 and the other at k_0/k_-.

For both cases, in the non-cooperative case with a fixed dissociation ratio the choice of this kinetic rate determines completely the relative weight of the modes as in (32). Cooperativity allows us to tune the weight of the mode corresponding to the bound state without changing the location as mentioned before.

Comparison with Fast Promoter Kinetics:
In order to demonstrate that slow switching is responsible for the emergence of new modes compared to the deterministic model, consider the non-cooperative self-regulating gene network (31) with fast promoter kinetics modeled by letting ε grow without bound in the first reversible TF-promoter binding/unbinding reactions. We state the following proposition which is proved in the Methods section:

Proposition SI-3. *As $\varepsilon \to \infty$, the stationary distribution of the network* (31) *is given by:*

$$\pi(m) = \lim_{t\to\infty} \Pr[X(t) = m] = \frac{\alpha_- w_m + \alpha m w_{m+1}}{\alpha m + \alpha_-},$$

where w_m satisfies the following recurrence relation:

$$w_{m+1} = \frac{((k_1 m + (\alpha_-/\alpha)k_0)(m + (\alpha_-/\alpha) + 1)}{k_-(m+1)(m + (\alpha_-/\alpha))^2} w_m, \ m \geq 0.$$

where w_0 is chosen to satisfy $\sum_{m=0}^{\infty} w_m = 1$.

Proof. A decomposition dual to Eq. (2) can be written and it can be noted that the fast matrix is block-diagonal with respect to $\mathrm{X} + \mathrm{D}_1$ which is the slow variable, while the fast variable is D_1.

Expanding asymptotically the stationary distribution in terms of ε, and taking the limit as ε goes to zero, we can find the distribution for the slow variable as follows:

$$\Pr[X + D_1 = m] = w_m,$$

where w_m satisfies the following recurrence relation:

$$w_{m+1} = \frac{((k_1 m + (\alpha_-/\alpha)k_0)(m + (\alpha_-/\alpha) + 1)}{k_-(m+1)(m + (\alpha_-/\alpha))^2} w_m, \ m \geq 0.$$

The joint distribution can be given as:

$$\Pr[X + D_1 = m, D_1 = 0] = w_m \frac{\alpha_-}{\alpha m + \alpha_-},$$
$$\Pr[X + D_1 = m, D_1 = 1] = w_m \frac{\alpha m}{\alpha m + \alpha_-}.$$

Hence we can compute the marginal density of X as follows:

$$\begin{aligned}\Pr[X = m] &= \Pr[X + D = m, D = 0] + \Pr[X + D = m + 1, D = 1] \\ &= \frac{\alpha_- w_m + \alpha m w_{m+1}}{\alpha m + \alpha_-}.\end{aligned}$$

□

Since the ratio w_{m+1}/w_m is a ratio of two polynomials and the denominator's degree is higher than the numerator, then stationary distribution is unimodal, while slow TF-gene binding/unbinding was shown to give a bimodal distribution (see (32)).

3.3 The Toggle Switch

A toggle switch is a basic GRN that exhibits deterministic multi-stability. It has two stable steady states and can switch between them with an external input or via noise. The ideal behavior is that only one gene is "on" at any moment in time. We now study the network with the slow promoter kinetics.

In the main text we have described a symmetric toggle switch. Here, we describe the general case. Consider the following network with cooperativity indices n, m:

$$\begin{aligned}
\mathrm{Y}_m + \mathrm{D}_0^X &\underset{\alpha_{-1}}{\overset{\alpha_1}{\rightleftharpoons}} \mathrm{D}_1^X & \mathrm{X}_n + \mathrm{D}_0^Y &\underset{\alpha_{-2}}{\overset{\alpha_2}{\rightleftharpoons}} \mathrm{D}_1^Y \\
\mathrm{D}_0 &\overset{k_{10}}{\rightarrow} \mathrm{D}_0^X + \mathrm{X}, & \mathrm{D}_0^Y &\overset{k_{20}}{\rightarrow} D_0^Y + \mathrm{Y}, \\
\mathrm{X} &\overset{k_{-1}}{\rightarrow} 0 & \mathrm{Y} &\overset{k_{-2}}{\rightarrow} 0 \\
n\mathrm{X} &\underset{\beta_{-1}}{\overset{\beta_1}{\rightleftharpoons}} \mathrm{X}_n & m\mathrm{Y} &\underset{\beta_{-2}}{\overset{\beta_2}{\rightleftharpoons}} \mathrm{Y}_m.
\end{aligned}$$

For the case $n, m = 1$, there is no multi-merization reaction. For consistency, we choose $\beta_1 = \beta_{-1}, \beta_2 = \beta_{-2} = 1$ in that case.

D^X, D^Y denote the states of the promoters of the two genes expressing X, Y, respectively.

Denote the promoter configuration species by D^X, D^Y. Then the network has four configurations $(\mathrm{D}^X, \mathrm{D}^Y) \in \{(0,0), (0,1)\ , (1,0), (1,1)\}$. Using Corollary 4 we expect to have a stationary distribution with four modes $(k_{10}/k_{-1}, k_{20}/k_{-2})$, $(k_{10}/k_{-1}, 0)$, $(0, k_{20}/k_{-2})$, $(0,0)$. Using the algorithm of Proposition SI-2, the reduced-order Markov chain infinitesimal generator is:

$$\Lambda_r = \begin{bmatrix} -\alpha_1\rho_2 - \alpha_2\rho_1 & \alpha_{-2} & \alpha_{-1} & 0 \\ \alpha_2\rho_1 & -\alpha_{-2} & 0 & \alpha_{-1} \\ \alpha_1\rho_2 & 0 & -\alpha_{-1} & \alpha_{-2} \\ 0 & 0 & 0 & -\alpha_{-2} - \alpha_{-1} \end{bmatrix}, \tag{35}$$

where

$$\rho_1 = \left(\frac{k_{10}}{k_{-1}}\right)^n \frac{\beta_1}{n!\beta_{-1}}, \quad \rho_2 = \left(\frac{k_{20}}{k_{-2}}\right)^m \frac{\beta_2}{m!\beta_{-2}}. \tag{36}$$

We notice immediately from the last row in the matrix (35) that the transition rates towards the configuration (1,1) are zero, which implies that the weight of the mode corresponding to $(1,1)$ is zero. Hence, we have three modes only. The weights corresponding to the modes can be found as the principal eigenvector of Λ_r as given in Corollary 4. Hence, the stationary distribution for X, Y is:

$$\pi(x,y) = \frac{1}{\frac{\alpha_1}{\alpha_{-1}}\rho_2 + \frac{\alpha_2}{\alpha_{-2}}\rho_1 + 1}\left(\mathbf{P}(y; \tfrac{k_{20}}{k_{-2}})\mathbf{P}(x; \tfrac{k_{10}}{k_{-1}}) + \frac{\alpha_1}{\alpha_{-1}}\rho_2\mathbf{P}(y; \tfrac{k_{20}}{k_{-2}})\delta(x) + \frac{\alpha_2}{\alpha_{-2}}\rho_1\mathbf{P}(x; \tfrac{k_{10}}{k_{-1}})\delta(y)\right). \tag{37}$$

Since the stationary distribution has three modes, it deviates from the ideal behavior of a switch where at most two stable steady states, under appropriate parameter conditions, are possible. Nevertheless, a bimodal distribution can be achieved by minimizing the weight of the first mode at $(\frac{k_{10}}{k_{-1}}, \frac{k_{20}}{k_{-2}})$. If we fix $\alpha_1/\alpha_{-1}, \alpha_2/\alpha_{-2}$, then this can be satisfied by tuning $n, m, \beta_{\pm 1}, \beta_{\pm 2}$ to maximize ρ_1, ρ_2 in (36). Choosing higher cooperativity indices, subject to $n < k_{10}/k_{-1}, m < k_{20}/k_{-2}$, achieves this.

The toggle switch has three modes regardless of the cooperativity index. This is unlike the deterministic model where only one positive stable state is realizable with non-cooperative binding, and two stable steady states are realizable with cooperative binding. However, the toggle switch with fast switching can admit three modes in some parameter ranges. In contrast to the case of slow switching under consideration here, the third stable state is the (low,low) state [12].

Monte-Carlo simulations via Gillespie's algorithm for the cooperative toggle switch have been performed to investigate the minimum time-scale separation to recover our predictions. We have chosen the parameters such that the (low,high), and (high,low) modes get 0.25 each, and the (high,high) modes gets 0.5 at the slow promoter limit. Figure A-a shows that the three modes predicted by the analysis have been recovered with promoter kinetics being just *two times* slower than the protein decay rate. Figure A-b shows a sample trajectory where the three modes are visible.

Alternative model of the toggle switch

Instead of modeling the toggle switch as two independent genes with their own promoters, an alternative model consists of a single promoter regulating two operons [13], [14]. In our modelling framework this amounts to a single gene expression block with two transcription factors and two

expressed proteins, i.e., two inputs and two outputs. Despite the fact that our formalism accounts for a single expressed protein, we find no difficulty in applying our methods as shown below.

The alternative model can be written as:

$$\begin{array}{llll} \mathrm{X}_c + \mathrm{D}_{00} \rightleftharpoons \mathrm{D}_{10}, & \mathrm{X}_c + \mathrm{D}_{01} \rightleftharpoons \mathrm{D}_{11}, & \mathrm{Y}_c + \mathrm{D}_{00} \rightleftharpoons \mathrm{D}_{01}, & \mathrm{Y}_c + \mathrm{D}_{10} \rightleftharpoons \mathrm{D}_{11}, \\ \mathrm{D}_{00} \xrightarrow{k_x} \mathrm{D}_{00} + \mathrm{X}, & \mathrm{D}_{00} \xrightarrow{k_y} \mathrm{D}_{00} + \mathrm{Y}, & \mathrm{D}_{10} \xrightarrow{k_x} \mathrm{D}_{10} + \mathrm{X}, & \mathrm{D}_{01} \xrightarrow{k_y} \mathrm{D}_{01} + \mathrm{Y}, \\ \mathrm{X} \xrightarrow{k_{-x}} \emptyset, & \mathrm{Y} \xrightarrow{k_{-y}} \emptyset, & n\mathrm{X} \underset{\beta_{-x}}{\overset{\beta_x}{\rightleftharpoons}} \mathrm{X}_c, & m\mathrm{Y} \underset{\beta_{-y}}{\overset{\beta_y}{\rightleftharpoons}} \mathrm{Y}_c, \end{array} \tag{38}$$

Assuming slow-promoter kinetics, the stationary distribution can be decomposed into a mixture of Poisson distributions centered at $(\frac{k_x}{k_{-x}}, \frac{k_y}{k_{-y}}), (\frac{k_x}{k_{-x}}, 0), (0, \frac{k_y}{k_{-y}})$. Despite the fact that model appears to be different, the resulting modes and the reduced-order Markov chain are similar to the model used before.

3.4 The Repressilator

The repressilator is a synthetic biological circuit that implements a ring oscillator. It is a canonical example of a GRN that exhibits a limit cycle, i.e. sustained oscillation. The list of reactions is given as follows:

$$\begin{array}{rlrlrl} \mathrm{Z}_n + \mathrm{D}_0^X & \underset{\alpha_-}{\overset{\alpha}{\rightleftharpoons}} \mathrm{D}_1^X & \mathrm{X}_n + \mathrm{D}_0^Y & \underset{\alpha_-}{\overset{\alpha}{\rightleftharpoons}} \mathrm{D}_1^Y & \mathrm{Y}_n + \mathrm{D}_0^Z & \underset{\alpha_-}{\overset{\alpha}{\rightleftharpoons}} \mathrm{D}_1^Z \\ \mathrm{D}_0 & \xrightarrow{k} \mathrm{D}_0^X + \mathrm{X}, & \mathrm{D}_0^Y & \xrightarrow{k} D_0^Y + \mathrm{Y}, & \mathrm{D}_0^Z & \xrightarrow{k} D_0^Z + \mathrm{Z}, \\ \mathrm{X} & \xrightarrow{k_-} 0 & \mathrm{Y} & \xrightarrow{k_-} 0 & \mathrm{Z} & \xrightarrow{k_-} 0 \\ n\mathrm{X} & \underset{\beta_-}{\overset{\beta}{\rightleftharpoons}} \mathrm{X}_n & n\mathrm{Y} & \underset{\beta_-}{\overset{\beta}{\rightleftharpoons}} \mathrm{Y}_n. & n\mathrm{Z} & \underset{\beta_-}{\overset{\beta}{\rightleftharpoons}} \mathrm{Z}_n. \end{array}$$

Deterministic analysis of the repressilator [15] reveals that it does not oscillate with non-cooperative binding. We proceed to apply our analysis methods to study the behavior in the stochastic case with slow promoter kinetics.

Denote the promoter configuration species by $\mathrm{D}^X, \mathrm{D}^Y, \mathrm{D}^Z$. Then the network has eight configurations $(\mathrm{D}^X, \mathrm{D}^Y, \mathrm{D}^Z) \in \{(0,0,0), (0,0,1)\ , ..., (1,1,1)\}$. Using Corollary 4 we expect to have a stationary distribution with eight modes. Using the algorithm of Proposition SI-2, the reduced-order Markov chain infinitesimal generator is:

$$\Lambda_r = \begin{bmatrix} -3\alpha\rho & \alpha_- & \alpha_- & 0 & \alpha_- & 0 & 0 & 0 \\ \alpha\rho & -\alpha_- - \alpha\rho & 0 & \alpha_- & 0 & \alpha_- & 0 & 0 \\ \alpha\rho & 0 & -\alpha_- - \alpha\rho & \alpha_- & 0 & 0 & \alpha_- & 0 \\ 0 & \alpha\rho & 0 & -2\alpha_- & 0 & 0 & 0 & \alpha_- \\ \alpha\rho & 0 & 0 & 0 & -\alpha_- - \alpha\rho & \alpha_- & \alpha_- & 0 \\ 0 & 0 & 0 & 0 & \alpha\rho & -2\alpha_- & 0 & \alpha_- \\ 0 & 0 & \alpha\rho & 0 & 0 & 0 & -2\alpha_- & \alpha_- \\ 0 & 0 & 0 & 0 & 0 & 0 & 0 & -3\alpha_-. \end{bmatrix}, \tag{39}$$

where $\rho = \left(\frac{k}{k_-}\right)^n \frac{\beta}{n!\beta_-}$.

The stationary distribution is:

$$\pi(x,y,z) = \frac{1}{D}\Big(w\mathbf{P}(x,y,z;K,0,0) + w\mathbf{P}(x,y,z;0,K,0) + w\mathbf{P}(x,y,z;0,0,K) \tag{40}$$
$$+\frac{1}{2w}\mathbf{P}(x,y,z;K,K,K) + \mathbf{P}(x,y,z;K,K,0) + \mathbf{P}(x,y,z;0,K,K) + \mathbf{P}(x,y,z;K,0,K)\Big),$$

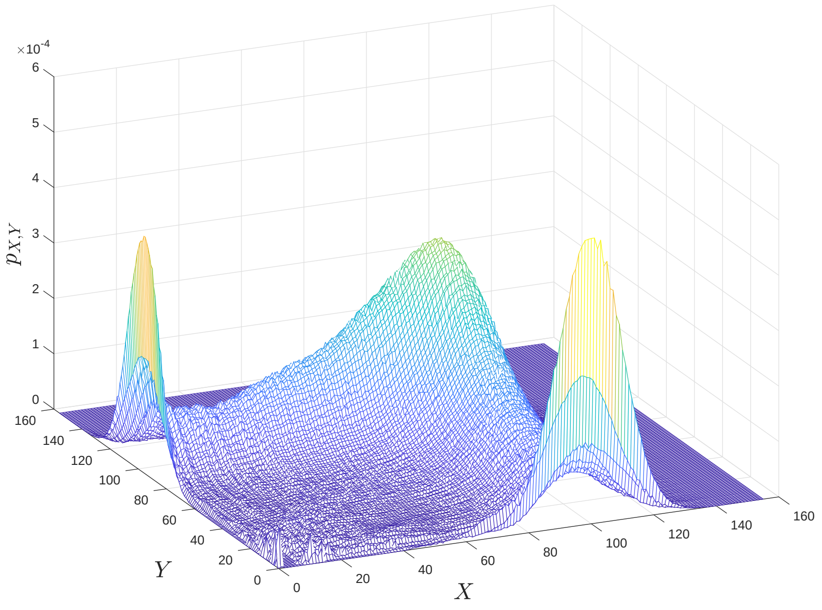

(a)

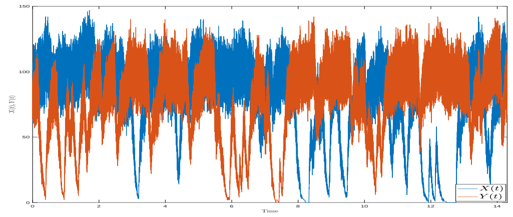

(b)

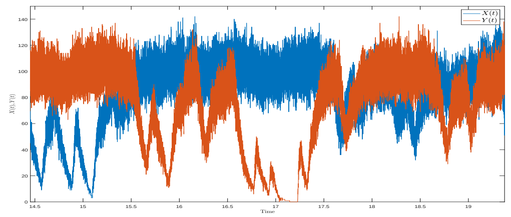

(c)

Figure A: **Analysis predicts the stationary distribution with a two-to-one time scale separation.** (a) The empirical stationary distribution for the toggle switch. It has been computed based on a realization of the stochastic process. The realization has been computed via Gillespie's algorithm and has been averaged over 70×10^6 iterations. (b) A sample trajectory of the toggle switch. (c) A zoomed plot showing the three modes. Observe that between times 18 and 18.5 both modes are high. The parameters are: $\alpha_- = 10, \alpha = 0.1, k_- = 20, k = 2000, \beta = 20, \beta_- = 2000$. Note that the unbinding rate is two times slower than the protein decay rate.

where $K = \frac{k}{k_-}, w = 2\rho\frac{\alpha}{\alpha_-}, D = 3w + 3 + \frac{1}{2w}$. Notice that the Poisson distribution centered at (0,0,0) has zero weight. Hence, the network can admit up to seven modes.

We will study the behavior of this network in two cases: when $w \gg 1$, and $w \approx 1$.

The "Stochastic Oscillator"

Although the distribution is a mixture of seven modes, note that it reduces to three effective modes if

$$w = 2\frac{\alpha}{\alpha_-}\left(\frac{k}{k_-}\right)^n \frac{\beta}{n!\beta_-} \gg 1,$$

where the three modes are located at $d_x = (K, 0, 0), d_y = (0, K, 0), d_z = (0, 0, K)$. The tri-modal stationary distribution is consistent with the classical oscillations of the repressilator, and this is independent of the cooperativity index. Note that this condition is analogous to the oscillation condition in the deterministic model [15] (but with cooperativity only) which also requires "large" production ratio.

In order to study whether the network oscillates, we need to define a notion of limit cycle for a stochastic system. Due to randomness, the time-series can not be periodic. Nevertheless, since the stationary distribution is tri-modal, we say that the network oscillates if the time trajectory continues to jump between the modes in the same order.

Assume $w \gg 1$. Let d_x, d_y, d_z be the three dominant modes. We will show that if the reduced-order Markov chain is at mode r_x then it is much more likely to transition to d_y rather than to d_z. Similar arguments apply if we start from d_y, d_z.

Let $Q(t) = e^{t\Lambda_r}$ be the probability transition matrix [2]. The (i, j) entry in the transition matrix denotes the probability of being at i at time t if we start from j. Hence, $Q_{ij}(t) = \Pr[D(t) = i|D(0) = j]$.

We are interested in comparing the probabilities of transiting from r_x to r_y, r_z. Hence we study small $t \ll 1$. We utilize the power series expansion to evaluate $Q(t)$. Let $\Lambda_d = \text{diag}(\Lambda_r)$ and $\Lambda_+ = \Lambda_r - \Lambda_d$, where $\text{diag}(\Lambda_r)$ is the diagonal matrix which contains the diagonal entries of Λ_r. Note that Λ_+ is positive. Since Λ_d and Λ_+ commute, we can write

$$Q(t) = e^{\Lambda_d}e^{\Lambda_+}.$$

We can approximate $Q(t)$ for small t by writing the Taylor expansion of e^{Λ_+}. Hence, using (39) we write a third-order Taylor series for the transition probabilities that we need:

$$\begin{aligned}
Q_{d_xd_x} &= e^{-2t} + e^{-2t}o(t) = e^{-2t}(1 + wt^2) + e^{-2t}o(t^3)\\
Q_{d_yd_x} &= e^{-2t}o(t) = e^{-2t}(\tfrac{1}{2}wt^2) + e^{-2t}o(t^3)\\
Q_{d_zd_x} &= e^{-2t}o(t^3),
\end{aligned}$$

where we assumed, w.l.o.g, that $\alpha_- = 1$.

Using the expressions above, if the Markov chain is at r_x then it is most likely to stay there. The transition is much more likely to happen to d_y rather than d_z. Hence, we expect to see "long" periods of protein X being expressed, and then it jumps to express protein Y, and then protein Z. Since the finite Markov chain is ergodic, the pattern repeats.

Note that the analysis above predicts that both the cooperative and the noncooperative repressilator are capable of oscillation with slow-promoter kinetics when $w \gg 1$.

Multi-modality in the repressilator with slow-promoter kinetics
In the case that w is close to 1, the seven modes in (40) share the probability almost equally. This is independent of the cooperativity index. Compare this the non-oscillatory deterministic model, which is mono-stable and it can't admit multiple stable equilibria.

3.5 Synchronization of interconnected toggle switches

We consider N identical toggle switches:

$$\begin{array}{cccccc}
\mathrm{Y}_{ic} + \mathrm{D}_0^{xi} & \underset{\varepsilon\alpha_{-x}}{\overset{\varepsilon\alpha_x}{\rightleftharpoons}} & \mathrm{D}_1^{xi} & \mathrm{X}_{ic} + \mathrm{D}_0^{yi} & \underset{\varepsilon\alpha_{-y}}{\overset{\varepsilon\alpha_y}{\rightleftharpoons}} & \mathrm{D}_1^{yi} \\
\mathrm{D}_0^{xi} & \overset{k_x}{\to} & \mathrm{X}_i + \mathrm{D}_0^{xi}, & \mathrm{D}_0^{yi} & \overset{k_y}{\to} & \mathrm{Y}_i + \mathrm{D}_0^{yi}, \\
\mathrm{X}_i & \overset{k_{-x}}{\to} & 0, & \mathrm{Y}_i & \overset{k_{-y}}{\to} & 0, \\
n\mathrm{X}_i & \underset{\beta_{-x}}{\overset{\beta_x}{\rightleftharpoons}} & \mathrm{X}_{ic}, & n\mathrm{Y}_i & \underset{\beta_{-y}}{\overset{\beta_y}{\rightleftharpoons}} & \mathrm{Y}_{ic},
\end{array}$$

where $i = 1, .., N$. Note that the model above is more general than the one considered in the main text which describes a population of symmetric toggle switches. We interconnect these systems through diffusion of the protein species $\mathrm{X}_i, \mathrm{Y}_i$ among cells, modeled through reversible reactions with a diffusion coefficient Ω:

$$\mathrm{X}_i \underset{\Omega}{\overset{\Omega}{\rightleftharpoons}} \mathrm{X}_j, \quad \mathrm{Y}_i \underset{\Omega}{\overset{\Omega}{\rightleftharpoons}} \mathrm{Y}_j, \quad i \neq j, \ i, j = 1, .., N. \tag{41}$$

For a deterministic model, there exists a parameter range for which all toggle switches will synchronize into bistability for sufficiently high diffusion coefficient [16]. This implies each switch in the network behaves as a bistable switch, and it converges with all the other switches to the same steady-states.

Our aim is to analyze the stochastic model at the limit of slow promoter kinetics and compare it with the deterministic model. The network has 4^N promoter configurations. Consider a promoter configuration

$$d = (d^X, d^Y) := (d_1^X, .., d_N^X, d_1^Y, ..., d_N^Y) \in \{0, .., 4^N - 1\},$$

where $d_i^X, d_j^Y \in \{0, 1\}$. As discussed before, we need to represent the Markov chain conditioned on $D(t) = d$. Each conditional Markov chain can be represented as follows:

$$\begin{array}{cccccc}
\emptyset & \underset{k_{-x}}{\overset{d_i^X k_x}{\rightleftharpoons}} & \mathrm{X}_i, & \emptyset & \underset{k_{-y}}{\overset{d_i^Y k_y}{\rightleftharpoons}} & \mathrm{Y}_i, \\
n\mathrm{X}_i & \underset{\beta_{-x}}{\overset{\beta_x}{\rightleftharpoons}} & \mathrm{X}_{ic}, & n\mathrm{Y}_i & \underset{\beta_{-y}}{\overset{\beta_y}{\rightleftharpoons}} & \mathrm{Y}_{ic},
\end{array}$$

$$\mathrm{X}_i \underset{\Omega}{\overset{\Omega}{\rightleftharpoons}} \mathrm{X}_j, \quad \mathrm{Y}_i \underset{\Omega}{\overset{\Omega}{\rightleftharpoons}} \mathrm{Y}_j, \quad i, j = 1, .., N.$$

This conditional Markov is not the forms (54), (55) that arise from the class of GRNs defined previously as in Figure 1. Nevertheless, it can be observed that it is a reversible zero-deficiency network [17]. We show in the SI-§4.1 that our results can be generalized to networks that admit weakly reversible deficiency zero conditional Markov chains.

As shown in the SI-§4.1, there are 4^N conditional Markov chains, and their stationary distributions are Poisson distributions with the following set of modes:

$$\left\{ \left(\frac{k_x}{k_{-x}} \omega_{d_1}^X, \ldots, \frac{k_x}{k_{-x}} \omega_{d_N}^X, \frac{k_y}{k_{-y}} \omega_{d_1}^Y, \ldots, \frac{k_y}{k_{-y}} \omega_{d_N}^Y \right) : d = (d^X, d^Y) \in \{0, .., 4^N - 1\} \right\}, \tag{42}$$

where

$$\omega_{d_i}^X = \frac{\sum_{i=1}^N \bar{d}_i^X + \bar{d}_i^X k_{-x}/\Omega}{N + k_{-x}/\Omega}, i = 1, .., N, \tag{43}$$

in which $\bar{d}_i^X = 1 - d_i^X$. $\omega_{d_i}^Y$ is defined similarly.

Using Theorem 3, the stationary distribution is a mixture of 4^N Poisson distributions, and the weights can be found by finding the principal eigenvector of the infinitesimal generator of the reduced-order Markov chain as before. Proposition SI-2, gives the procedure to find the matrix Λ_r, where Eq. (27) is replaced by (47) with (51). As before, the Markov states are the configurations $d = (d^X, d^Y) \in \{0, .., 4^N - 1\}$. Assume that $D(t) = d$. Then, the state transitions are given by the following reactions:

$$\mathrm{D}_0^{xi} \underset{\alpha_{-x}}{\overset{\alpha_x \rho_{d_i}^X}{\rightleftharpoons}} \mathrm{D}_1^{xi}, \quad \mathrm{D}_0^{yi} \underset{\alpha_{-y}}{\overset{\alpha_y \rho_{d_i}^Y}{\rightleftharpoons}} \mathrm{D}_1^{yi}, \tag{44}$$

where

$$\rho_{d_i}^X = \mathbb{E}[\mathrm{X}_{ic} | D(t) = d] = \frac{1}{n!} \frac{\beta_x}{\beta_{-x}} \left(\frac{k_x}{k_{-x}} \omega_{d_i}^X \right)^n, \tag{45}$$

and $\rho_{d_i}^Y$ is defined analogously.

Note that the mode corresponding to the state in which all the TFs are bound to the promoters has no incoming transitions in the reduced Markov chain and hence it has zero weight, hence the network has $4^N - 1$ modes.

We consider now the case of a high diffusion coefficient. Note from (42), (43), (45) that as $\Omega \to \infty$, $X_1, .., X_N$ will synchronize in the sense that the joint distribution of $X_1, .., X_N$ is symmetric with respect to all permutations of the random variables. This implies that the marginal stationary distributions $p_{X_i}, i = 1, .., N$ are identical. Hence, for sufficiently large Ω the probability mass is concentrated around the region for which $X_1, .., X_N$ are close to each other. Consequently, for large Ω we can replace the population of toggle switches with a *single toggle switch* with the *synchronized protein processes* $X(t), Y(t)$, which are defined, for the sake of convenience, as $X(t) := X_1(t), Y(t) := Y_1(t)$. Next, we describe the stationary distribution of $X(t), Y(t)$.

From (43) it can be seen that $\omega_{d_i}^X$ does not depend on d_i for large Ω. Instead it depends only on $\sum_{i=1}^N \bar{d}_i^X$, which is the total number of unbound promoter sites in the genes producing $\mathrm{X}_1, .., \mathrm{X}_N$. The same holds for $\omega_{d_i}^Y$. Hence, the number of modes will drop from $4^N - 1$ to $(N+1)^2 - 1$. Hence the joint distribution of X, Y is a mixture of Poisson distributions with the following modes:

$$\left\{ \left(\frac{ik_x}{Nk_{-x}}, \frac{jk_y}{Nk_{-y}} \right) : i, j = 0, .., N, (i, j) \neq (0, 0) \right\}.$$

Note that similar to the single toggle switch, there are modes which have both X, Y with non-zero copy number. On the other hand, there are many additional modes. Recall that in the case of a single toggle switch, we have tuned the cooperativity ratios such that the modes in which both genes are ON are suppressed. Similarly, the undesired modes can be suppressed by tuning the cooperativity ratio which can be achieved by choosing $\rho_{d_i}^X, \rho_{d_i}^Y, d = 0, .., 4^N - 1$ sufficiently large. In particular, letting the multi-merization ratio $\beta_x/\beta_{-x}, \beta_y/\beta_{-y} \to \infty$, the weights of modes in the interior of the positive orthant $\mathbb{R}_+^2$ approach zero.

In conclusion, for sufficiently high Ω and sufficiently high multimerization ratio the population behaves as a *multimodal switch* , which means that the whole network can have either the gene X ON, or the gene Y ON. And every gene can take $2N$ modes which are:

$$\left\{ \left(\frac{ik_x}{Nk_{-x}}, 0 \right), \left(0, \frac{ik_y}{Nk_{-y}} \right) : i = 1, .., N \right\}.$$

Comparing to the low diffusion case, the network will have up to $2^N - 1$ modes with sufficiently high multimerization ratio.

Comparison with the deterministic model For Ω greater then a certain threshold, the deterministic system bifurcates into bistabiliy. This means that all toggle switches converge to the same exact equilibria if Ω is greater than the threshold. As we have seen before, this is not the case for the stochastic system, since the toggle switches converges *asymptotically* to each other. Hence, we need to choose a a threshold for Ω that constitutes "sufficient" synchronization. We choose to define this as the protein processes synchronizing within one copy number. In other words, we require the maximum distance between the modes in (42) to be less than 1.

This amounts to requiring the diffusion coefficient needs to satisfy:

$$\Omega \geq \frac{1}{N} \max\{k_x - k_{-x}, k_y - k_{-y}\}. \tag{46}$$

We derive (46) below.
In order for the maximum distance between the modes in (42) to be less than 1, we need to satisfy this inequality:

$$\max_{d \in \{0,..,4^{n-1}\}} \max_{i,j=1,..,N} \left\{ \left| \frac{k_x}{k_{-x}} \omega^X_{d_i} - \frac{k_x}{k_{-x}} \omega^X_{d_j} \right|, \left| \frac{k_y}{k_{-y}} \omega^Y_{d_i} - \frac{k_y}{k_{-y}} \omega^Y_{d_j} \right| \right\} < 1.$$

Let us consider first maximizing the term containing the variables related to the gene X. Note from (43) that $\omega^X_{d_i}$ and $\omega^X_{d_j}$ can differ only in a k_{-x}/Ω term in the numerator. Hence, the maximization can be simplified to:

$$\max \left\{ \frac{k_x}{k_{-x}} \left(\frac{k_{-x}/\Omega}{N + k_{-x}/\Omega} \right), \frac{k_y}{k_{-y}} \left(\frac{k_{-y}/\Omega}{N + k_{-y}/\Omega} \right) \right\} < 1.$$

Solving for Ω yields (46).

4 Extension to GRNs with Complex-Balanced Conditional Markov Chains

In the main text we have included assumptions to simplify the mathematical treatment and notations while retaining the same qualitative features of the problem. In this section, we elucidate the manner in which the results can be generalized.

The class of networks defined in the main text does not allow direct interactions between the proteins. However, our main results hold for a more general class of networks. Figure 2 depicts a generalized gene expression block where the proteins expressed by other genes participate in the gene reactions block. The gene expression block remains unchanged. The basic theory presented in the main text (Proposition 1, Theorem 3) remains unchanged. Proposition SI-2 needs can be modified by replacing Eq. (27) by the following:

$$[\Lambda_r]_{d'd} = \begin{cases} \alpha, & \text{if the reaction is monomolecular} \\ \alpha \mathbb{E}[X_{\bar{i}c} | D = d], & \text{if the reaction is bimolecular} \end{cases} \tag{47}$$

However, since there are no closed form formulae for computing the conditional expectation in general, the expression above is of limited utility. Nevertheless, we can define a more general

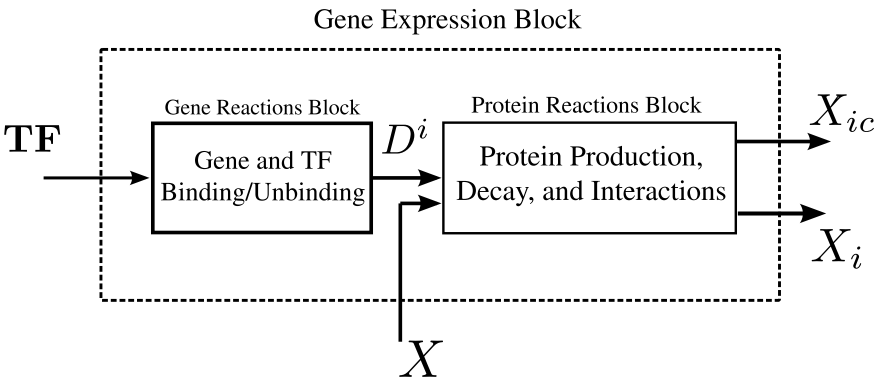

Figure B: A generalized representation of the gene expression block. X represent the vector of all protein monomers in the network.

class of network such that the conditional stationary distributions can be computed analytically as obtained in Proposition 2. In order to define our generalized class of networks, we need some further notation and background which are summarized in the following subsection.

4.1 Complex-Balancing and the Concept of Deficiency

Recall that a reaction network consists of the set of species $\mathscr{S}$ and the set of reactions $\mathscr{R}$. A reaction $\mathrm{R}_j \in \mathscr{R}$ can be written as Eq. (1).

The formal linear combination in the left-side of the reaction is called the *reactant complex*, while the linear combination on the right-side of the reaction is called the *product complex*. The set of all complexes in the network is denoted by $\mathscr{C}$. Each complex can be interpreted as a vector belonging to the Euclidean vector space whose unit vectors correspond to the species.

Define the following matrix:

$$\tilde{\Gamma} = [A|B], \text{ where } [A]_{ij} = \alpha_{ij}, [B]_{ij} = \beta_{ij},$$

where α_{ij}, β_{ij} are the stoichiometry coefficients. If we remove the redundant columns of $\tilde{\Gamma}$, this yields an $|\mathscr{S}| \times |\mathscr{C}|$ matrix Γ_c, where each column corresponds to a certain complex. Hence, it can be verified that there exists a matrix $Y \in \{0, \pm 1\}^{|C| \times |\mathscr{R}|}$ such the that the stoichiometry matrix Γ admits the following factorization:

$$\Gamma = \Gamma_c Y. \tag{48}$$

There is significant literature studying the reaction networks using the notion of complexes, initially for deterministic systems, [18],[17], and then for stochastic systems [19],[20]. The ordinary differential equation for a reaction network can be written as:

$$\dot{x} = \Gamma R(x), \tag{49}$$

where x is the concentration vector, and R is a reaction rate function which is commonly defined using mass-action kinetics as follows:

$$R_j(x) = k_j \prod_{i=1}^{|\mathscr{S}|} x_i^{a_{ji}},$$

where k_j is reaction rate constant. A conservation law is a nonnegative vector v such that $v^T \Gamma = 0$. The stoichiometric compatibility class containing x_0 is defined as $(x_\circ + \Im\Gamma) \cap \mathbb{R}^n_{\geq 0}$. If there are no conservation laws then $\mathbb{R}^n_{\geq 0}$ is a stoichiometric compatibility class.

In order to state the subsequent results, we define a *modified* mass-action kinetics. For a given a reaction rate function R, the modified reaction rate function is given as

$$\tilde{R}_j(x) = \tilde{k}_j \prod_{i=1}^{|\mathscr{S}|} x_i^{a_{ji}}, \text{ with } \tilde{k}_j := \frac{k_j}{\prod_{i=1}^{|\mathscr{S}|} a_{ji}!}.$$

As per (48), the complex formation rate is defined as $R_c(x) := YR(x)$. An equilibrium $\hat{x}$ of (49) is called *complex balanced* if it satisfies $R_c(x) = 0$ also. The existence of a single complex-balanced equilibrium guarantees that all equilibria of the network are complex balanced [18].

Physically, reaction rates in $\ker Y$ do not change the complex-formation rate. Hence, if $\ker \Gamma = \ker Y$, then every equilibrium is complex-balanced. This ensures that a network is complex balanced regardless of kinetic rate constants. Hence, the *deficiency* of the network is defined as $\delta := \dim(\ker(\Gamma_c) \cap \text{Image}(Y))$. If $\delta = 0$ then every equilibrium is complex-balanced, and the network is said to be a zero-deficiency network.

A network is weakly reversible if: existence of a directed path from complex C_i to complex C_j, implies the existence a directed path from C_j to C_i. This is equivalent to the existence of a strictly positive vector in $\ker Y$.

For weakly-reversible networks, a simple graphical characterization of the deficiency is given by [17]:

$$\delta = |\mathscr{C}| - \ell - \text{rank}(\Gamma), \tag{50}$$

where ℓ is the number of strongly connected components in the graph of complexes.

A main result in the theory of deterministic complex-balanced networks is stated as:

Lemma SI-1 ([17]). *If a reaction network with mass-action kinetics is weakly reversible and has deficiency zero, then there exists a unique positive equilibrium in every stoichiometric compatibility class.*

Hence if the network is complex-balanced and without conservation laws, then it has a unique equilibrium for the deterministic system. A parallel result exists for the Markov chain model with associated chemical master equation. It is stated as follows:

Lemma SI-2 ([19],[20]). *Assume a network is weakly reversible, has deficiency zero and has no conservation laws. Let $\hat{x} = [\hat{x}_1, .., \hat{x}_n]^T$ be the unique complex balance equilibrium for the deterministic system with the modified mass-action kinetics. Then the stationary distribution for the corresponding master equation is:*

$$\pi(x) = \prod_{i=1}^{n} \mathbf{P}(x_i; \hat{x}_i).$$

4.2 Extension of the Main Result

We are ready to define the generalized class of networks as gene regulatory networks whose conditional Markov chains are complex-balanced. In particular, if all the conditional Markov chains are weakly reversible and zero-deficiency, then the GRN can be treated with our methods.

Consider a set of N genes, binding sets $\{B_i\}_{i=1}^N$, and kinetics constants k_j. A *generalized gene expression block* is as shown in Figure B. Then, a gene regulatory network is an arbitrary interconnection of gene expression blocks subject to Assumption A2. A gene regulatory network admits conditional product-form distributions if, for each $d \in \{0, .., L-1\}$, the reaction network corresponding to the conditional Markov chain is weakly reversible and deficiency zero. Hence, we can restate Proposition 2:

Proposition SI-4. *Fix $d \in \{0,..,L-1\}$. Let $\hat{x}^d, \hat{x}^{d2}$ be the complex-balanced equilibria for the proteins and multi-merized proteins, respectively. Consider Eq. (4), then there exists a conditional joint stationary distribution $\pi^{(J)}_{X|d}$ and it is given by*

$$\pi^{(J)}_{X|d}(x) = \prod_{i=1}^{N} \pi_{X|di}(x_i), \tag{51}$$

where

$$\pi^{(J)}_{X|d_i}(x_i) = \begin{cases} \mathbf{P}\left(x_{i1}, x_{i2}; \hat{x}^d_i, \hat{x}^{d2}_i\right) & \text{if } X_i \text{ is multimerized} \\ \mathbf{P}\left(x_i; \hat{x}^d_i\right), & \text{otherwise} \end{cases}, \tag{52}$$

where (J) refers to the joint distribution in multimerized and non-multimerized processes, x_{i1} refers to the copy number of X_i, while x_{i2} refers to the copy number of X_{ic}, $\mathbf{P}(x;a) := \frac{a^x}{x!}e^{-a}$, $\mathbf{P}(x_1, x_2; a_1, a_2) := \frac{a_1^{x_1}}{x_1!}\frac{a_2^{x_2}}{x_2!}e^{-a_1-a_2}$.

4.3 Specific Examples

4.3.1 Cooperative Binding

Cooperative binding is the process in which a TF forms as a hetero-dimer consisting of two different proteins. Consider two proteins X, Y, then the hetero-dimerization reaction:

$$\mathrm{X} + \mathrm{Y} \leftrightarrow \mathrm{XY}.$$

Consider a conditional Markov chain that consists of uncoupled birth-death processes, with multi-merization reactions. It is reversible and has deficiency zero. If a hetero-dimerization reaction is added, then this involves adding two complexes, adding a new strongly connected component to the complexes' graph, and adding a new linearly-independent row to the stoichiometry matrix. According to (50) this will not change the deficiency. Hence, our results can be extended immediately to networks with cooperative binding.

4.3.2 Diffusion

We model diffusion between two gene expression blocks as a reversible reaction between the proteins as:

$$\mathrm{X} \leftrightarrow \mathrm{Y}.$$

Consider a conditional Markov chain that consists of uncoupled birth-death processes, with multi-merization reactions. It is reversible and has deficiency zero. If a diffusion reaction is added, then none of the number of complexes, the number of strongly connected components, or the rank of the stoichiometry matrix change. According to (50) this will not change the deficiency. Hence, our results can be extended immediately to networks with diffusion. We have already applied this to the communicating toggle switches in the main text.

4.3.3 Multi-Step Multi-merization

In the main text we have assumed that the multi-merization process occurs in one-step only as follows:

$$nX \underset{\beta_-}{\overset{\beta}{\rightleftharpoons}} X_n. \tag{53}$$

As an alternative model, consider the following multi-step or *sequential* multi-merization process:

$$2X \underset{\beta_{-2}}{\overset{\beta_2}{\rightleftharpoons}} X_2$$

$$X_2 + X \underset{\beta_{-3}}{\overset{\beta_3}{\rightleftharpoons}} X_3$$

$$\vdots$$

$$X_{n-1} + X \underset{\beta_{-n}}{\overset{\beta_n}{\rightleftharpoons}} X_n$$

Consider a conditional Markov chain, conditioned on $D = d$, that consists of the above network with a birth and death process:

$$0 \underset{k_-}{\overset{k}{\rightleftharpoons}} X.$$

In order to evaluate the deficiency (50) of the network, note that $|\mathscr{C}| = 2+2n, \ell = n+1, \mathrm{rank}(\Gamma) = n+1$. Hence, $\delta = 0$. Therefore, the network is complex balanced and the stationary distribution is a product of Poisson distributions.

Since X_n acts as a transcription factor we are interested in finding $\mathbb{E}[X_n|D = d]$. Using Lemma SI-2, we can find the required quantity by finding the a complex-balanced equilibrium of the associated deterministic system with modified mass-action kinetics. Hence, we find:

$$\mathbb{E}[X_n|D = d] = \frac{1}{2}\left(\frac{k}{k_-}\right)^n \prod_{i=2}^{n} \frac{\beta_i}{\beta_{-i}}.$$

From the perspective of the slow promoter kinetics, the multi-step multi-merization reactions can be replaced with a single reaction (53) with $\beta/\beta_- = \frac{1}{2}n! \prod_{i=2}^{n} \beta_i/\beta_{-i}$.

5 Extension to Networks with Multiple Copies of the Genes

In the main text, we have assumed that there exists one copy only per gene. Nevertheless, our theory is not limited by this assumption. In this subsection we show how the theory can be generalized.

Consider the network in Figure 2. Assume that the ith gene has M_i copies. Let B_i be the corresponding binding-set, and denote $b_i = |B_i| - 1$. We need to track the promoter configurations for all copies of the ith gene. Hence, we define the *total binding set* P_i. The new set P_i describes all possible $|B_i|$-tuples of nonnegative integers such that they sum to M_i. It can be shown that $|P_i| = \binom{M_i+b_i}{b_i}$. To explain this, consider the following:

- If one TF binds to a promoter or the promoter changes its configuration autonomously, then $B_i = \{0, 1\}$, while $P_i := \{(0, M_i), (1, M_i), .., (M_i, 0)\}$. Hence $|P_i| = M_i + 1$.

- If two promoters bind independently to a promoter then $B_i = \{00, 01, 10, 11\}$, while $P_i := \{(M_i, 0, 0, 0), (M_i - 1, 1, 0, 0), ..., (0, 0, 0, M_i)\}$, and $|P_i| = (M_i + 3)(M_i + 2)(M_i + 1)/6$. Each tuple $(d_{i0}, .., d_{i3})$ is interpreted as follows: there are d_{i0} copies of the gene at configuration 00, d_{i1} at configuration 01, etc. The tuple entries are ordered to correspond to 00,01,10,11, respectively.

- If two TFs bind competitively to a promoter then the binding set is $B_i := \{00, 10, 01\}$, while $P_i := \{(M_i, 0, 0), (M_i - 1, 1, 0), ..., (0, 0, M_i)\}$, and $|P_i| = (M_i + 2)(M_i + 1)/2$. The tuple entries are ordered to correspond to 00,01,10, respectively.

Consider the master equation (3). Let us consider that the ith gene has M_i copies. We again split the stochastic process $Z(t)$ into two subprocesses: *the gene process* $D(t)$ and *the protein process* $X(t)$. We will redefine $D(t)$ as explained below.

Consider the ith gene. We define the process D_i such that $D_i(t) \in P_i$, so it informs us exactly the copy numbers of genes at each promoter configuration. Collecting these into a vector, define the gene process $D(t) := [D_1(t), ..., D_N(t)]^T$ where $D(t) \in \prod_{i=1}^N P_i$. The ith gene can be represented by $|P_i|$ states, so $L := \prod_{i=1}^N |P_i|$ is the total number of configurations in the GRN. With abuse of notation, we write also $D(t) \in \{0, .., L-1\}$ in the sense of some fixed bijection between $\{0, .., L-1\}$ and $\prod_{i=1}^N P_i$. Hence, $d \in \{0, .., L-1\}$ corresponds to $(d_1, ..., d_N) \in P_1 \times .. \times P_N$ and we write $d = (d_1, .., d_N)$. Note that $d_i = (d_{i0}, .., d_{ib_i})$. With the redefined D, the protein processes and the master equation are defined in an identical manner to the main text.

In order to apply Proposition 2, we need to reexamine the conditional Markov chains. Fix $D(t) = d$, and consider the ith gene. Let $d_i = (d_{i0}, .., d_{ib_i})$, which informs us the copy number of genes with a certain configuration. Hence, the production rate for the protein is the sum of the production rates of the genes weighted by their respective copy numbers. Then, the conditional Markov chain is given as:

$$\emptyset \underset{k_{-i}}{\overset{k_{id_i}}{\rightleftharpoons}} \mathrm{X}_i, \tag{54}$$

or, with multimerization, as:

$$\emptyset \underset{k_{-i}}{\overset{k_{id_i}}{\rightleftharpoons}} \mathrm{X}_i, \;\; n_i\mathrm{X}_i \underset{\beta_{-i}}{\overset{\beta_i}{\rightleftharpoons}} \mathrm{X}_{ic}. \tag{55}$$

where

$$k_{id_i} := \sum_{j=0}^{b_i} d_{ij} k_{ij}. \tag{56}$$

Theorem 3 and Corollary 4 hold as in the main text. Hence we conclude that

$$\pi(x) = \sum_{d=0}^{L-1} \lambda_d \pi_{X|d}(x) = \sum_{d=0}^{L-1} \lambda_d \prod_{i=1}^{N} \mathbf{P}\left(x_i; \frac{k_{id_i}}{k_{-i}}\right). \tag{57}$$

In order to find the weights λ_i's, we need to restate Proposition SI-2 as follows:

Proposition SI-5. *The matrix Λ_r in eq. (24) can be computed via the algorithm below.*

- *For each $d \in \{0, .., L-1\}$ write $d = (d_1, .., d_N) = ((d_{10}, ..., d_{1b_1}), .., (d_{N0}, ..., d_{Nb_N})) \in \prod_{i=1}^N P_i$. Using the previously discussed identification:*
  - *Let $\mathscr{R}_d = \{\mathrm{R}_1, .., \mathrm{R}_{|\mathscr{R}_d|}\}$ be the set of all gene reactions. Then, for each $\nu \in \{1, .., |\mathscr{R}_d|\}$:*
    - *1. Let D_j^i, and $\mathrm{D}_{j'}^i$ be the reactant and product configuration species of the R_ν, where $j \in B_i$. The reaction will cause a transition from d to a state d' in which $d'_{ij} = d_{ij} - 1$ $d'_{ij'} = d_{ij'} + 1$, and $d'_{ij} = d_{ij}$ otherwise. Let α be the kinetic constant of R_ν. If R_ν is a binding reaction, then let $\mathrm{X}_{\bar{i}}$, $\mathrm{X}_{\bar{i}c}$ denote the TF or the multi-merized TF, where $\bar{i}$ denotes the index of the gene that expresses the TF.*

      2. Then, the (d', d) entry of Λ_r can be written as:

$$[\Lambda_r]_{d'd} = \begin{cases} \alpha, & \textit{if the reaction is monomolecular} \\ \frac{\alpha}{n_{\bar{i}}!} \frac{\beta_{\bar{i}}}{\beta_{-\bar{i}}} \left(\frac{k_{\bar{i}d_{\bar{i}}}}{k_{-\bar{i}}}\right)^{n_{\bar{i}}}, & \textit{if the reaction is bimolecular} \end{cases} \tag{58}$$

where $k_{\bar{i}d_{\bar{i}}}$ is defined in (56).

- *Set*

$$[\Lambda_r]_{dd} = -\sum_{i' \neq i} \mathbf{1}^T \hat{\Lambda}_{d_{i'} d_i} \pi_d. \tag{59}$$

- *Set the rest of the entries of Λ_r to zero.*

Example: The Self-Regulating Gene
Let us consider again the non-cooperative self-regulating gene,

$$\begin{aligned} \mathrm{X} + \mathrm{D}_0 &\underset{\varepsilon\alpha_-}{\overset{\varepsilon\alpha}{\rightleftharpoons}} \mathrm{D}_1 \\ \mathrm{D}_0 &\overset{k_0}{\rightarrow} \mathrm{D}_0 + \mathrm{X}, \\ \mathrm{D}_1 &\overset{k_1}{\rightarrow} \mathrm{D}_1 + \mathrm{X}, \\ \mathrm{X} &\overset{k_-}{\rightarrow} 0. \end{aligned}$$

Assume that the gene has M copies. Hence, the total binding set $P_i = \{(M,0)..., (0,M)\}$, and we have $M+1$ states. Using Proposition 5 the reduced order Markov chain can be represented as follows:

$$(M,0) \underset{\alpha_-}{\overset{\alpha M k_0/k_-}{\rightleftharpoons}} (M-1,1) \underset{\alpha_-}{\overset{\alpha(k_1+(M-1)k_0)/k_-}{\rightleftharpoons}} (M-2,2) \ldots \underset{\alpha_-}{\overset{\alpha M k_1 k_0/k_-}{\rightleftharpoons}} (0,M).$$

The resulting stationary distribution is a mixture of Poissons with modes at $Mk_0, (M-1)k_0 + k_1, ..., Mk_1$. Figure C shows the stationary distribution for several values of M for the network above. The number of modes increases with M, however, the stationary distribution converges for high M.

6 Additional Information for Tables and Figures in the Main Text

6.1 Figure 1-b

A self-repressing gene is given as:

$$\begin{aligned} \mathrm{X} + \mathrm{D}_0 &\underset{\varepsilon\alpha_-}{\overset{\varepsilon\alpha}{\rightleftharpoons}} \mathrm{D}_1 \\ \mathrm{D}_0 &\overset{k_0}{\rightarrow} \mathrm{D}_0 + \mathrm{X}, \\ \mathrm{X} &\overset{k_-}{\rightarrow} 0. \end{aligned} \tag{60}$$

The slow kinetic limit is calculated via (32), while the fast kinetics limit is calculated by iterating the recurrence relation in Proposition SI-2. The remaining curves are computed by a finite projection solution [21] of the CME truncated at $x = 65$. This amounts to writing the master equation as a finite linear differential equation of the form:

$$\dot{p} = \Lambda p,$$

then evaluating the principal eigenvector of Λ using the command `null` in MATLAB, and normalizing the resulting vector.

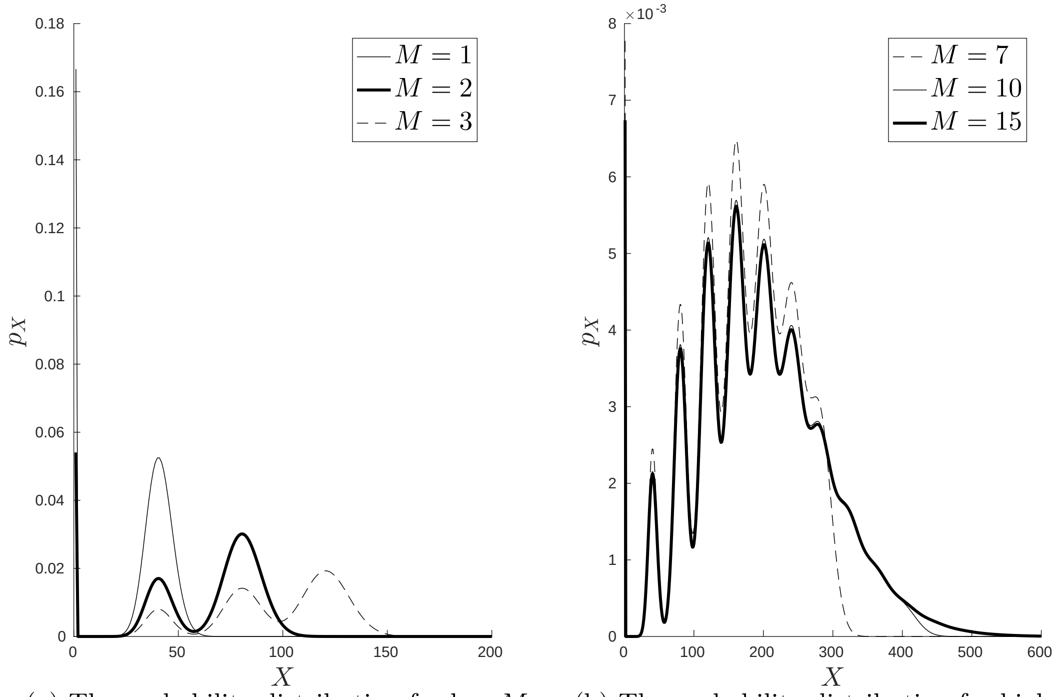

(a) The probability distribution for low M (b) The probability distribution for high M

Figure C: **The number of modes increase with the number of gene copies.** (a) The stationary distribution for one, two and three copy numbers. The parameters are chosen to be identical to the one in Figure 1. (b) The stationary distribution for seven, ten and fifteen. Note coefficients of the high modes are very small.

The ordinary differential equations for the deterministic model can be written as:

$$\begin{bmatrix} \dot{d}_0 \\ \dot{d}_1 \\ \dot{x} \end{bmatrix} = \begin{bmatrix} -1 & 1 & 0 & 0 \\ 1 & -1 & 0 & 0 \\ -1 & 1 & 1 & -1 \end{bmatrix} \begin{bmatrix} \alpha d_0 x \\ \alpha_- d_1 \\ k_0 d_0 \\ k_- x \end{bmatrix},$$

with $d_0(t) + d_1(t) = 1$ for all t. Setting the derivatives to zero, and solving for x gives the following characteristic equation:

$$\alpha k_- x^2 + \alpha_- k_- x - \alpha_- k_0 = 0.$$

Since the third term has a negative sign, the network has a unique positive equilibrium for all parameter values. The parameters used in the simulation are:

$$\alpha = \varepsilon/200, \alpha_- = \varepsilon, k_0 = 40, k_- = 1.$$

The deterministic equilibrium is $\hat{x} \approx 34.1641$. The curve for the fast promoter kinetic has been computed by applying the recurrence relation in Proposition 3.

6.2 Figure 1-d

A hybrid repression-activation network is given as:

$$\begin{array}{lll@{\qquad}lll} \mathrm{Y} + \mathrm{D}_0^X & \underset{\alpha_{-1}}{\overset{\alpha_1}{\rightleftharpoons}} & \mathrm{D}_1^X & \mathrm{X} + \mathrm{D}_0^Y & \underset{\alpha_{-2}}{\overset{\alpha_2}{\rightleftharpoons}} & \mathrm{D}_1^Y \\ \mathrm{D}_0 & \overset{k_{10}}{\to} & \mathrm{D}_0^X + \mathrm{X}, & \mathrm{D}_1^Y & \overset{k_{21}}{\to} & \mathrm{D}_1^Y + \mathrm{Y}, \\ \mathrm{X} & \overset{k_{-1}}{\to} & 0 & \mathrm{Y} & \overset{k_{-2}}{\to} & 0. \end{array}$$

The parameters used in the simulation are:

$$\alpha_1 = \varepsilon/70, \alpha_{-1} = \varepsilon, \alpha_2 = \varepsilon/20, \alpha_{-2} = \varepsilon, k_{10} = k_{21} = 40, k_{-1} = k_{-2} = 1.$$

Modelling the network deterministically, there exists a unique positive equilibrium where the equilibrium values of X, Y are given as $\hat{x} \approx 25.4727, \hat{y} \approx 39.9216$. The stationary distribution has been computed using Corollary 4 and Proposition SI-2.

6.3 Table 1

Let us consider the non-cooperative self-regulating gene. The reaction network is given as:

$$\begin{aligned} \mathrm{X} + \mathrm{D}_0 & \underset{\varepsilon\alpha_-}{\overset{\varepsilon\alpha}{\rightleftharpoons}} \mathrm{D}_1 \\ \mathrm{D}_0 & \overset{k_0}{\to} \mathrm{D}_0 + \mathrm{X}, \\ \mathrm{D}_1 & \overset{k_1}{\to} \mathrm{D}_1 + \mathrm{X}, \\ \mathrm{X} & \overset{k_-}{\to} 0. \end{aligned} \tag{61}$$

The deterministic model gives the following characteristic equation for x:

$$\alpha k_- x^2 + (\alpha_- k_- - \alpha k_1) x - \alpha_- k_0 = 0.$$

Since the third term has a negative sign, the network has a unique positive equilibrium for all parameter values. For the stochastic model: With no leakiness, i.e. $k_0 = 0$, the Markov chain has an absorbing state at $(X, D_1) = (0, 0)$. Hence, the network has a single mode at zero. With leakiness, we have shown in the main text that the slow promoter kinetics give rise to two modes (Corollary 4). While for fast promoter kinetics, we have mentioned that it gives rise to a uni-modal distribution after Proposition 3. Let us consider the cooperative self-regulating gene. The reaction network is given as:

$$\begin{aligned} \mathrm{X}_c + \mathrm{D}_0 &\underset{\varepsilon\alpha_-}{\overset{\varepsilon\alpha}{\rightleftharpoons}} \mathrm{D}_1 \\ \mathrm{D}_0 &\xrightarrow{k_0} \mathrm{D}_0 + \mathrm{X}, \qquad (62) \\ \mathrm{D}_1 &\xrightarrow{k_1} \mathrm{D}_1 + X, \\ \mathrm{X} &\xrightarrow{k_-} 0 \\ 2\mathrm{X} &\underset{\varepsilon\beta_-}{\overset{\varepsilon\beta}{\rightleftharpoons}} \mathrm{X}_c \qquad (63) \end{aligned}$$

The deterministic model gives the following characteristic equation for x:

$$-\alpha\beta k_- x^3 + \alpha\beta k_1 x^2 - \alpha_-\beta_- k_- x + \alpha_-\beta_- k_0 = 0. \qquad (64)$$

Since the constant term is negative, this means that (64) has at least one positive real solution. The equation (64) has three solutions if

$$\alpha_1 k_1 k_-^2 \beta \alpha_{-1}\beta_{-1}(k_1 + 18k_0) > 4\alpha_1^2 k_1^3 \beta^2 k_0 + 27\alpha_1 k_-^2 \beta k_0^2 \alpha_{-1}\beta_{-1} + 4k_-^4 \alpha_{-1}^2 \beta_{-1}^2,$$

and one solution otherwise. Using the Routh-Hurwitz criterion, all solutions have positive real parts if and only if $k_1 > k_0$. This means that the network can have either one or two positive *stable* equilibria only.

For the stochastic model: with no leakiness, the discussion is identical to the previous case. With leakiness, we have shown in the main text that the slow promoter kinetics give rise to two modes independent of the cooperativity index (Corollary 4). While for fast promoter kinetics, a single mode has been verified by solving the master equation numerically for many parameter sets including the ones that are multi-stable for the deterministic system.

6.4 Figure 4b-e

We consider a population of three toggle switches ($N = 3$) with $k_x = k_y = 150, k_{-x} = k_{-y} = 1, \beta_x = \beta_y = 1, \beta_{-x} = \beta_{-y} = 1, \alpha_x = \alpha_y = 0.3\varepsilon, \alpha_{-x} = \alpha_{-y} = \varepsilon$ and $n = 2$.

The surface plots have been computed using Eq. (10).

6.5 Cell-Fate Circuit Reactions and Figure 6b-c

The first network has independent cooperative binding of the TFs to the promoters. So it can be written as follows [22]:

$$
\begin{array}{llll}
\mathrm{X}_2+\mathrm{D}^X_{00} \rightleftharpoons \mathrm{D}^X_{10}, & \mathrm{X}_2+\mathrm{D}^X_{01} \rightleftharpoons \mathrm{D}^X_{11}, & \mathrm{D}^X_{00} \overset{k_{x0}}{\to} \mathrm{D}^X_{00}+\mathrm{X}, & \mathrm{D}^X_{01} \overset{k_{x1}}{\to} \mathrm{D}^X_{01}+\mathrm{X}, \\
\mathrm{Y}_2+\mathrm{D}^X_{00} \rightleftharpoons \mathrm{D}^X_{01}, & \mathrm{Y}_2+\mathrm{D}^X_{10} \rightleftharpoons \mathrm{D}^X_{11}, & \mathrm{D}^X_{10} \overset{k_{x2}}{\to} \mathrm{D}^X_{10}+\mathrm{X}, & \mathrm{D}^X_{11} \overset{k_{x3}}{\to} \mathrm{D}^X_{11} \overset{k_{x3}}{\to} \mathrm{D}^X_{11}+\mathrm{X}, \\
\mathrm{X}_2+\mathrm{D}^Y_{00} \rightleftharpoons D^Y_{10}, & \mathrm{X}_2+\mathrm{D}^Y_{01} \rightleftharpoons \mathrm{D}^Y_{11}, & \mathrm{D}^Y_{00} \overset{k_{y0}}{\to} \mathrm{D}^Y_{00}+\mathrm{Y}, & \mathrm{D}^Y_{01} \overset{k_{y1}}{\to} \mathrm{D}^Y_{01}+\mathrm{Y}, \\
\mathrm{Y}_2+\mathrm{D}^Y_{00} \rightleftharpoons \mathrm{D}^Y_{01}, & \mathrm{Y}_2+\mathrm{D}^Y_{10} \rightleftharpoons \mathrm{D}^Y_{11}, & \mathrm{D}^Y_{10} \overset{k_{y2}}{\to} \mathrm{D}^Y_{10}+\mathrm{Y}, & \mathrm{D}^Y_{11} \overset{k_{y3}}{\to} \mathrm{D}^Y_{11} \overset{k_{y3}}{\to} \mathrm{D}^Y_{11}+\mathrm{Y}, \\
2\mathrm{X} \underset{\beta_{-x}}{\overset{\beta_x}{\rightleftharpoons}} \mathrm{X}_2, & 2\mathrm{Y} \underset{\beta_{-y}}{\overset{\beta_y}{\rightleftharpoons}} \mathrm{Y}_2 & \mathrm{X} \overset{k_{-x}}{\to} \emptyset, & \mathrm{Y} \overset{k_{-y}}{\to} \emptyset.
\end{array}
\tag{65}
$$

In order for the genes to be cross-inhibiting and self-activating we let: $k_{x1} = k_{y2} = 0$. Also, $k_{x2} > k_{x0}, k_{x3}$ and $k_{y1} > k_{y0}, k_{y3}$.

The second network that we study is a model of the PU.1/GATA.1 network, which is a lineage determinant in hematopoietic stem cells. The PU.1/GATA.1 network can be written as follows:

$$
\begin{array}{llll}
\mathrm{X}_2+\mathrm{D}^X_{00} \underset{\alpha_{-x0}}{\overset{\alpha_{x0}}{\rightleftharpoons}} \mathrm{D}^X_{10}, & \mathrm{Y}_2+\mathrm{D}^X_{10} \underset{\alpha_{-x1}}{\overset{\alpha_{x1}}{\rightleftharpoons}} \mathrm{D}^X_{11}, & \mathrm{D}^X_{00} \overset{k_{x0}}{\to} \mathrm{D}^X_{00}+\mathrm{X}, & \mathrm{D}^X_{10} \overset{k_{x1}}{\to} \mathrm{D}^X_{10}+\mathrm{X}, \\
\mathrm{Y}_2+\mathrm{D}^Y_{00} \underset{\alpha_{-y0}}{\overset{\alpha_{y0}}{\rightleftharpoons}} \mathrm{D}^Y_{01}, & \mathrm{X}_2+\mathrm{D}^Y_{01} \underset{\alpha_{-y1}}{\overset{\alpha_{y1}}{\rightleftharpoons}} \mathrm{D}^Y_{11}, & \mathrm{D}^Y_{00} \overset{k_{y0}}{\to} \mathrm{D}^Y_{00}+\mathrm{Y} & \mathrm{D}^Y_{01} \overset{k_{y1}}{\to} \mathrm{D}^Y_{01}+\mathrm{Y}, \\
\mathrm{X} \overset{k_{-x}}{\to} \emptyset, & \mathrm{Y} \overset{k_{-y}}{\to} \emptyset, & &
\end{array}
\tag{66}
$$

The parameters for Figure 5b are as follows: The dissociation ratio is fixed at 1/2000 for all binding/unbinding reactions and we let the dimerization ratio be 1/90. The production ratios are $k_{x2}/k_{-x} = k_{y1}/k_{-y} = 2700$. We assume that the inhibition and activation actions allow for leaks and hence we let $k_{x0}/k_{-x} = k_{y0}/k_{-y} = 1080$, $k_{x3}/k_{-x} = k_{y3}/k_{-y} = 675$ and $k_{x2}/k_{-x} = k_{y1}/k_{-y} = 20$.

The parameters for Figure 5c are as follows: The dissociation ratios are $\alpha_{x0}/\alpha_{-x0} = 10, \alpha_{x1}/\alpha_{-x1} = 1/2700, \alpha_{y0}/\alpha_{-y0} = 10, \alpha_{y1}/\alpha_{-x1} = 1/2700$. The maximal production ratio is $k_{x1}/k_{-x} = k_{y1}/k_{-y} = 2700$, while $k_{x0}/k_{-x}27, k_{x2}/k_{-y} = 0$, $k_{y2}/k_{-y} = 0, k_{y0}/k_{-y} = 270$.

We ratio of the production to the leak were chosen as $k_{x1} : k_{x0} \approx 100 : 1$, and $k_{y1} : k_{y0} \approx 5{-}10 : 1$ [23],[24].

References

1. David F Anderson and Thomas G Kurtz. *Stochastic analysis of biochemical systems.* Springer, 2015.

2. James R Norris. *Markov Chains.* Cambridge University Press, 1998.

3. RZ Khasminskii, G Yin, and Q Zhang. Constructing asymptotic series for probability distributions of Markov chains with weak and strong interactions. *Quarterly of Applied Mathematics*, 55(1):177–200, 1997.

4. Arjun Raj, Charles S Peskin, Daniel Tranchina, Diana Y Vargas, and Sanjay Tyagi. Stochastic mRNA synthesis in mammalian cells. *PLoS Biol*, 4(10):e309, 2006.

5. Hong Qian, Pei-Zhe Shi, and Jianhua Xing. Stochastic bifurcation, slow fluctuations, and bistability as an origin of biochemical complexity. *Physical Chemistry Chemical Physics*, 11(24):4861–4870, 2009.

6. Andrew Duncan, Shuohao Liao, Tomáš Vejchodskỳ, Radek Erban, and Ramon Grima. Noise-induced multistability in chemical systems: Discrete versus continuum modeling. *Physical Review E*, 91(4):042111, 2015.

7. Srividya Iyer-Biswas and Ciriyam Jayaprakash. Mixed poisson distributions in exact solutions of stochastic autoregulation models. *Physical Review E*, 90(5):052712, 2014.

8. Vahid Shahrezaei and Peter S Swain. Analytical distributions for stochastic gene expression. *Proceedings of the National Academy of Sciences*, 105(45):17256–17261, 2008.

9. Shangying Wang. *Quantifying gene regulatory networks.* PhD thesis, Duke University, April 2014.

10. JEM Hornos, D Schultz, GCP Innocentini, JAMW Wang, AM Walczak, JN Onuchic, and PG Wolynes. Self-regulating gene: an exact solution. *Physical Review E*, 72(5):051907, 2005.

11. Melissa Vellela and Hong Qian. A quasistationary analysis of a stochastic chemical reaction: Keizer's paradox. *Bulletin of mathematical biology*, 69(5):1727–1746, 2007.

12. Rui Ma, Jichao Wang, Zhonghuai Hou, and Haiyan Liu. Small-number effects: a third stable state in a genetic bistable toggle switch. *Physical review letters*, 109(24):248107, 2012.

13. Patrick B Warren and Pieter Rein Ten Wolde. Enhancement of the stability of genetic switches by overlapping upstream regulatory domains. *Physical review letters*, 92(12):128101, 2004.

14. Aryeh Warmflash, Prabhakar Bhimalapuram, and Aaron R Dinner. Umbrella sampling for nonequilibrium processes. *The Journal of chemical physics*, 2007.

15. Domitilla Del Vecchio and Richard M Murray. *Biomolecular Feedback Systems.* Princeton Univ Press, 2015.

16. Evgeni V Nikolaev and Eduardo D Sontag. Quorum-sensing synchronization of synthetic toggle switches: A design based on monotone dynamical systems theory. *PLoS Comput Biol*, 12(4):e1004881, 2016.

17. Martin Feinberg. Chemical reaction network structure and the stability of complex isothermal reactors—I. the deficiency zero and deficiency one theorems. *Chemical Engineering Science*, 42(10):2229–2268, 1987.

18. Fritz Horn and Roy Jackson. General mass action kinetics. *Archive for rational mechanics and analysis*, 47(2):81–116, 1972.

19. David F Anderson, Gheorghe Craciun, and Thomas G Kurtz. Product-form stationary distributions for deficiency zero chemical reaction networks. *Bulletin of mathematical biology*, 72(8):1947–1970, 2010.

20. Eduardo D Sontag and Doron Zeilberger. A symbolic computation approach to a problem involving multivariate poisson distributions. *Advances in Applied Mathematics*, 44(4):359–377, 2010.

21. Slaven Peleš, Brian Munsky, and Mustafa Khammash. Reduction and solution of the chemical master equation using time scale separation and finite state projection. *The Journal of Chemical Physics*, 125(20):204104, 2006.

22. H Feng and J Wang. A new mechanism of stem cell differentiation through slow binding/unbinding of regulators to genes. *Scientific reports*, 2(2):550, 2012.

23. Keizo Nishikawa, Makoto Kobayashi, Atsuko Masumi, Susan E Lyons, Brant M Weinstein, P Paul Liu, and Masayuki Yamamoto. Self-association of Gata1 enhances transcriptional activity in vivo in zebra fish embryos. *Molecular and cellular biology*, 23(22):8295–8305, 2003.

24. Yutaka Okuno, Gang Huang, Frank Rosenbauer, Erica K Evans, Hanna S Radomska, Hiromi Iwasaki, Koichi Akashi, Francoise Moreau-Gachelin, Youlin Li, Pu Zhang, et al. Potential autoregulation of transcription factor PU. 1 by an upstream regulatory element. *Molecular and cellular biology*, 25(7):2832–2845, 2005.
